# Experimental Relocation of the Mitochondrial *ATP9* Gene to the Nucleus Reveals Forces Underlying Mitochondrial Genome Evolution

**DOI:** 10.1371/journal.pgen.1002876

**Published:** 2012-08-16

**Authors:** Maïlis Bietenhader, Alexandre Martos, Emmanuel Tetaud, Raeka S. Aiyar, Carole H. Sellem, Roza Kucharczyk, Sandra Clauder-Münster, Marie-France Giraud, François Godard, Bénédicte Salin, Isabelle Sagot, Julien Gagneur, Michelle Déquard-Chablat, Véronique Contamine, Sylvie Hermann-Le Denmat, Annie Sainsard-Chanet, Lars M. Steinmetz, Jean-Paul di Rago

**Affiliations:** 1 Université Bordeaux, IBGC, UMR5095 CNRS, Bordeaux, France; 2 CNRS, IBGC, UMR5095 CNRS, Bordeaux, France; 3 Genome Biology Unit, European Molecular Biology Laboratory (EMBL), Heidelberg, Germany; 4 Université Paris-Sud, Centre de Génétique Moléculaire, UPR3404, CNRS, Gif-sur-Yvette, France; 5 Université Paris-Sud, Institut de Génétique et Microbiologie, UMR 8621, Orsay, France; 6 CNRS, Orsay, France; 7 Ecole Normale Supérieure, Paris, France; Max Planck Institute for Biology of Aging, Germany

## Abstract

Only a few genes remain in the mitochondrial genome retained by every eukaryotic organism that carry out essential functions and are implicated in severe diseases. Experimentally relocating these few genes to the nucleus therefore has both therapeutic and evolutionary implications. Numerous unproductive attempts have been made to do so, with a total of only 5 successes across all organisms. We have taken a novel approach to relocating mitochondrial genes that utilizes naturally nuclear versions from other organisms. We demonstrate this approach on subunit 9/c of ATP synthase, successfully relocating this gene for the first time in any organism by expressing the *ATP9* genes from *Podospora anserina* in *Saccharomyces cerevisiae*. This study substantiates the role of protein structure in mitochondrial gene transfer: expression of chimeric constructs reveals that the *P. anserina* proteins can be correctly imported into mitochondria due to reduced hydrophobicity of the first transmembrane segment. Nuclear expression of *ATP9*, while permitting almost fully functional oxidative phosphorylation, perturbs many cellular properties, including cellular morphology, and activates the heat shock response. Altogether, our study establishes a novel strategy for allotopic expression of mitochondrial genes, demonstrates the complex adaptations required to relocate *ATP9*, and indicates a reason that this gene was only transferred to the nucleus during the evolution of multicellular organisms.

## Author Summary

The mitochondrion, centre of cellular energy production, is derived from an ancient alpha-proteobacterium. While the vast majority of its genes have been transferred to the nuclear genome during evolution, a handful of genes remain in all mitochondrial genomes for reasons that are not fully understood. To investigate the evolutionary implications of gene transfer and evaluate potential treatments for the severe diseases caused by mitochondrial mutations, researchers have been attempting to express these genes from the nucleus for decades. In this study, we successfully relocate the mitochondrial gene *ATP9* to the nucleus for the first time. This gene encodes an extremely hydrophobic protein that, when relocated, is particularly challenging to import into mitochondria. We achieve this in baker's yeast by replacing *ATP9* with a naturally nuclear version from another fungal species. The “hybrid” mitochondria effectively produce energy and resemble normal mitochondria in many aspects, although they have some difficulty importing and assembling the foreign protein. Our findings elucidate the complex cellular and protein structure adaptations required for successful mitochondrial gene transfer, either during evolution or experimentally. This study thus delivers insight into the evolution of mitochondrial genomes and energy production.

## Introduction

Mitochondrial genomes are remnants of an ancestral prokaryotic genome that has been considerably reduced in size during the evolution of eukaryotes because of a massive loss of redundant information and gene transfer to the nucleus. Today, the main function of mitochondrial genomes is the production of a few proteins involved in oxidative phosphorylation. It is not fully understood why the mitochondrial genome has been maintained throughout two billion years of evolution. There are only two genes always found in mitochondrial genomes: those encoding cytochrome *b* and Cox1p, subunits of respiratory complexes III and IV respectively [1]. The other genes can be found in either mitochondrial or nuclear DNA, and sometimes in both genomes like *ATP9*, which encodes subunit 9/*c* of ATP synthase [2] (this protein is called Atp9p in yeast; in other organisms we will henceforth refer to it as subunit 9). Several hypotheses have been proposed to account for the retention of DNA in mitochondria. One theory is that the transfer of genes from mitochondria to the nucleus is still underway [3]. Alternatively, genes may have been confined to mitochondria due to the difficulty of transporting their protein products into the organelle, an idea that stems from the enrichment of genes encoding hydrophobic proteins in organellar genomes [4]. Other views suggest that rather than being trapped in organelles, genes may have been preferentially maintained there to adjust gene expression according to the redox [5]–[7] or metabolic [8] state of mitochondria; this could ensure optimal energy transduction while minimizing the production of harmful reactive oxygen species.

An apt organism for assessing these theories is the yeast *Saccharomyces cerevisiae*, a facultative aerobe where both the nuclear and mitochondrial genomes can be easily manipulated. This organism can therefore be used to test allotopic expression, *i.e.*, whether a mitochondrial gene can be functionally transferred to the nucleus. This assay involves inactivating the mitochondrial gene by mutation and testing a nuclear version of that gene for its capacity to restore respiratory function. This test has been carried out for five yeast proteins, two hydrophilic: bI4, an intron-encoded RNA maturase [9], and Var1p, a subunit of the mitochondrial ribosome [10], and three hydrophobic: the Atp8p subunit of mitochondrial ATP synthase with one transmembrane domain [11], the Cox2p subunit of respiratory complex IV (cytochrome *c* oxidase) which has two transmembrane segments [12], and the cytochrome *b* subunit of respiratory complex III, which is a highly hydrophobic protein with eight transmembrane segments [4]. In the cases of bI4, Var1p and Atp8p, adding a mitochondrial targeting sequence (MTS) and adjusting the genetic code were sufficient for functional allotopic expression of these proteins from nuclear DNA. The relocation of *COX2* required a minimal change to the structure of the protein: a single tryptophan to arginine substitution within a transmembrane segment. Conversely, attempts to relocate the cytochrome *b* gene have been entirely unsuccessful. Altogether, these findings do not refute the hypothesis that mitochondrial gene transfer may still be underway, but they also support the theory that some genes face an insurmountable barrier to functional relocation, such as the cytochrome *b* gene which is universally present in mitochondrial genomes.

In this study, our aim was to allotopically express the *ATP9* gene in *S. cerevisiae*. The protein it encodes has only two transmembrane segments, yet is extremely hydrophobic and classified as a proteolipid because it can easily be extracted from mitochondria with organic solvents [13]. Subunit 9 is present in several copies (ten in yeast [14]), forming a ring structure that is an essential component of the ATP synthase proton-translocating domain (F_O_). During proton transport, the subunit 9-ring rotates, resulting in conformational changes that favour the production of ATP by the catalytic head of the ATP synthase (F_1_) and its release into the mitochondrial matrix [15], [16].

As *ATP9* is naturally present in the nuclear DNA of a number of organisms, including filamentous fungi [2] and most animals [17], it is clear that there is no insurmountable barrier to functional relocation of this gene from mitochondria to the nucleus. A previous study has shown that the yeast Atp9p fused to a MTS can be imported and processed *in vitro* by mitochondria isolated from wild-type yeast [18], but to date there exists no description of a successful *in vivo* experiment. Here we take a novel approach that achieves allotopic expression of subunit 9 in yeast by using naturally nuclear genes from the filamentous fungus *Podospora anserina*. We use an array of techniques to demonstrate that substantial adaptations, including a reduction in hydrophobicity, are required for functional nuclear expression of this protein.

## Results

### Nuclear Versions of the Yeast Mitochondrial *ATP9* Gene Fail to Rescue *Δatp9* Yeast

**Figure 1:**
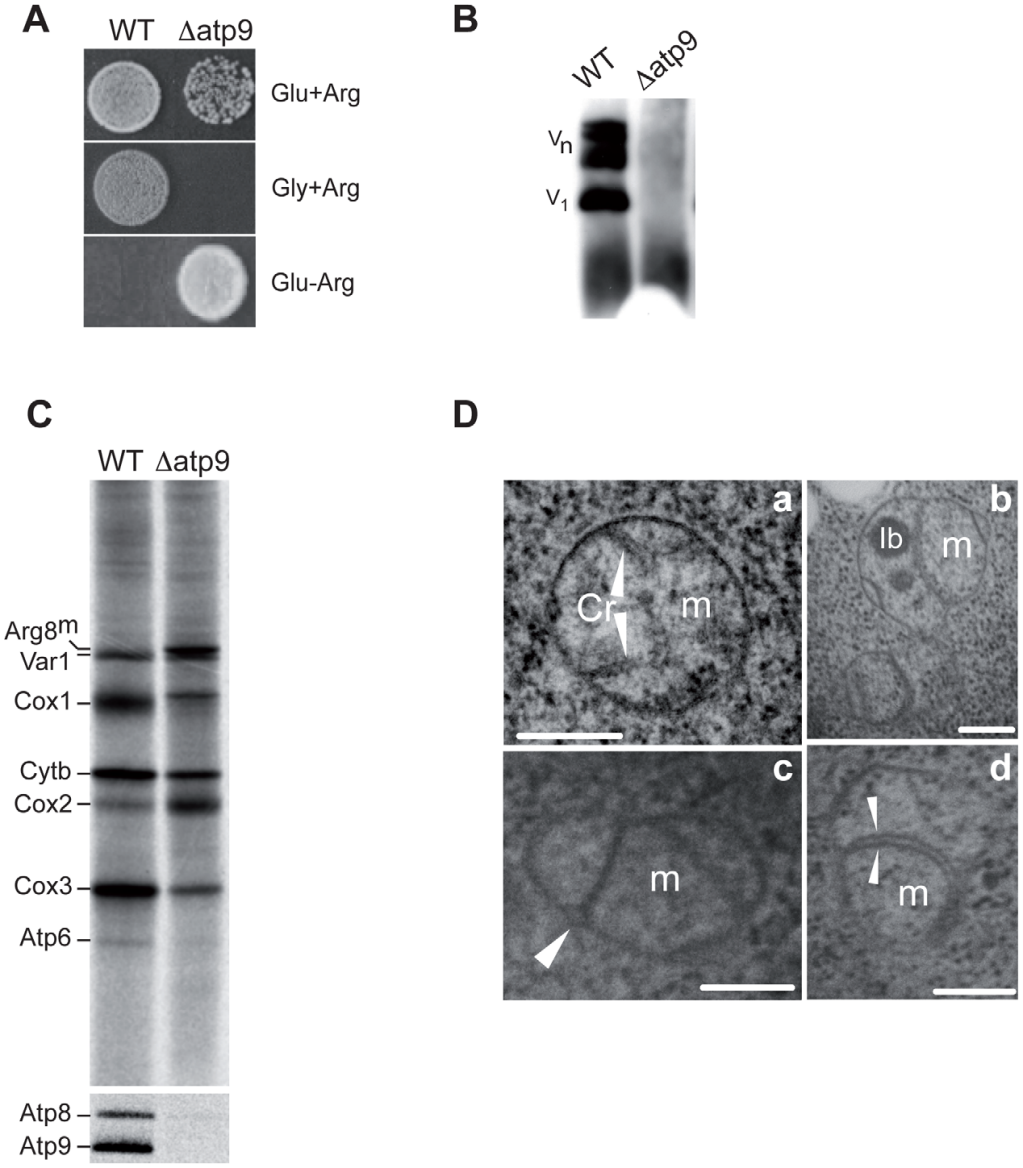
Deletion of the yeast mitochondrial *ATP9* gene and resulting phenotypes. A) The mitochondrial *ATP9* gene was deleted and replaced with *ARG8^m^* (see Figure S1 for details) in a wild-type strain lacking the nuclear *ARG8* gene. As a result, the *Δatp9* yeast grow on glucose (Glu) media lacking arginine (Arg) whereas the parental strain (*WT*) does not; in addition, *Δatp9* yeast cannot grow on glycerol (Gly). B) ATP synthase levels in *WT* and *Δatp9*. Isolated mitochondria were separated by BN-PAGE and western blotted with antibodies against Atp4p; *V*_1_ and *V*_n_ respectively indicate monomeric and oligomeric forms of ATP synthase. C) Pulse labelling of proteins translated in mitochondria. Total proteins were prepared from cells incubated in the presence of ^35^S methionine and cysteine as well as cycloheximide to inhibit cytosolic protein synthesis. Proteins (40,000 cpm per lane) were separated on 12% (Cox3p and Atp6p) or 17% (Atp9p and Atp8p) SDS-PAGE containing 6 M urea. D) Electron microscopy of *WT* (*a*) and *Δatp9* (*b–d*) cells grown in galactose (80 nm-thin sections); *m*, mitochondria; *Cr*, cristae; *Ib*, inclusion bodies; arrowheads in (*a*) point to *Cr*, to outer mitochondrial membrane in (*c*), and to septae in (*d*); *bars*, 0.2 µm.

As a first step in its nuclear relocation, we deleted the *ATP9* gene from the yeast mitochondrial genome using the *ARG8^m^* selection marker [19]. This was performed in a Δ*arg8* derivative of W303-1B, henceforth designated as *WT* (strain MR6 [20], all genotypes in Table 1). The *Δatp9* strain was thus selected for its capacity to grow in the absence of external arginine (Figure 1A) and proper integration of *ARG8^m^* at the *ATP9* locus was verified (Figure S1). As expected, *Δatp9* yeast is unable to grow on glycerol (Figure 1A) and devoid of assembled ATP synthase (Figure 1B). As has previously been observed in other yeast ATP synthase mutants, the *Δatp9* yeast also displayed several additional mitochondrial defects: (i) deficient synthesis of several mitochondrially-encoded proteins (Cox1p, Atp6p and Atp8p, Figure 1C); (ii) dramatically reduced amounts of respiratory complexes III and IV (<10% of *WT* levels, Figure S1E); (iii) aberrations in mitochondrial morphology including the absence of cristae and the presence of numerous septae within the mitochondrial matrix (Figure 1D); and (iv) decreased mtDNA stability leading to increased production of cytoplasmic *petites* resulting from large (>50%) deletions (ρ^−^) or complete loss (ρ^0^) of mtDNA (50–70% *vs.* <5% in *WT*) (see [20] and [21] and references therein for explanations of these secondary consequences).

**Table 1:** Genotypes and sources of yeast strains.

Strain	Nuclear genotype	mtDNA	Source
DFS160	*Matα Δleu2 ura3-52 ade2-101* Δ*arg8::URA3 kar1-1*	ρ^0^	[19]
DFS160-a	*Mata Δleu2 ura3-52 ade2-101* Δ*arg8::URA3 kar1-1*	ρ^0^	[51]
NB40-3C	*Mata lys2 leu2-3,112 ura3-52 his3ΔHinDIII arg8::hisG*	ρ^+^ *cox2-62*	[19]
SDC17-31b	*Matα met6 lys2 his3 ura3* Δ*arg8::hisG atp16::KanMX atp4::URA3*	ρ^+^ *Arg8^m^*	[52]
YTMT2	*Matα ade2 leu2-3,112 ura3-52 Δarg8::URA3*	ρ^+^ *cox2-62*	This study
FY1679	*MATa/MATα ura3-52/ura3-52; trp1Δ 63/TRP1; leu2Δ 1/LEU2; his3Δ 200/HIS3; GAL2/GAL2*	ρ^+^	[53]
MR6 (WT)	*Mata ade2-1 his3-11,15 trp1-1 leu2-3,112 ura3-1 CAN1* Δ*arg8::HIS3*	ρ^+^	[20]
MR6-*α*	*Matα ade2-1 his3-11,15 trp1-1 leu2-3,112 ura3-1 CAN1* Δ*arg8::HIS3*	ρ^+^	This study
RKY4	*Matα leu2Δ ura3-52 ade2-101 arg8::URA3 kar1-1*	ρ^−^ *Δatp9::ARG8^m^*	This study
RKY26 (*Δatp9*)	*Mata ade2-1 his3-11,15 trp1-1 leu2-3,112 ura3-1 CAN1* Δ*arg8::HIS3*	ρ^+^ *Δatp9::ARG8^m^*	This study
FG9	*Mata leu2Δ ura3-52 ade2-101 arg8::URA3 kar1-1*	ρ^−^ *ATP9*	This study
MB2	*Matα ade2-1 his3-11,15 trp1-1 leu2-3,112 ura3-1 CAN1* Δ*arg8::HIS3*	ρ^−^ *ATP9*	This study
AMY7	RKY26 transformed with plasmid pAM16 (*Δatp9*+*PaAtp9-5*, CEN)	ρ^+^ *Δatp9::ARG8^m^*	This study
AMY8	RKY26 transformed with plasmid pAM17 (*Δatp9*+*PaAtp9-7*, CEN)	ρ^+^ *Δatp9::ARG8^m^*	This study
AMY10	RKY26 transformed with plasmid pAM19 (*Δatp9*+*PaAtp9-5*, 2 µ)	ρ^+^ *Δatp9::ARG8^m^*	This study
AMY11	RKY26 transformed with plasmid pAM20 (*Δatp9*+*PaAtp9-7*, 2 µ)	ρ^+^ *Δatp9::ARG8^m^*	This study
AMY5	RKY26 transformed with plasmid pAM11 (*Δatp9*+*yAtp9-Nuc*, 2 µ*)*	ρ^+^ *Δatp9::ARG8^m^*	This study
AMY6	RKY26 transformed with plasmid pAM12 (*Δatp9*+*Atp9*-*Hyb*, 2 µ*)*	ρ^+^ *Δatp9::ARG8^m^*	This study

**Figure 2:**
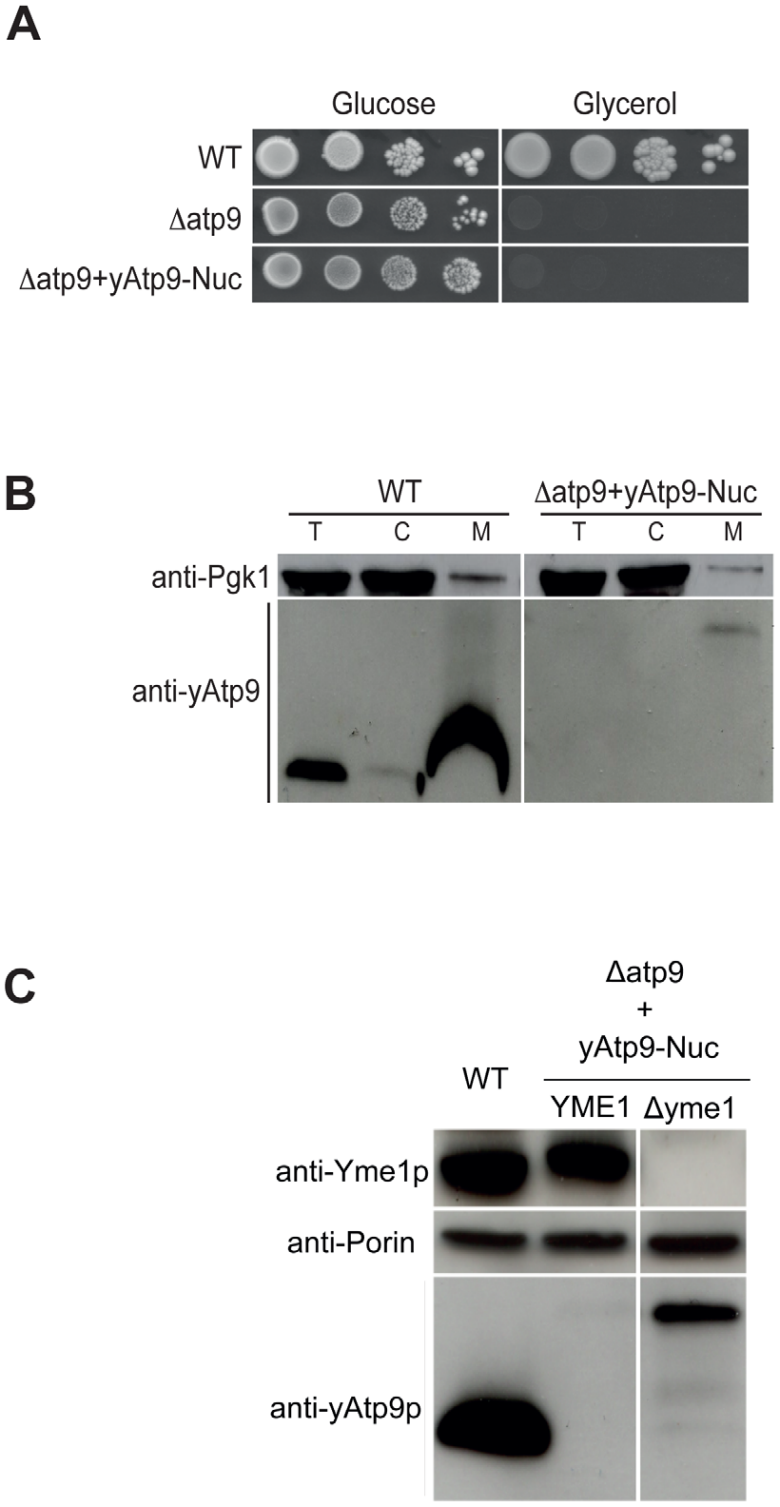
A nuclear version of the yeast mitochondrial *ATP9* gene fails to complement the *Δatp9* yeast. We engineered a nuclear version of the yeast *ATP9* gene (yAtp9-Nuc) by adding a mitochondrial targeting sequence (derived from the *P. anserina Atp9-7* gene) and adjusting the genetic code for nuclear expression of the endogenous gene (see Figure S2 and S3A for amino acid and nucleotide sequences). yAtp9-Nuc was tested for its capacity to complement *Δatp9* yeast with respect to respiratory capacity. A) Growth on rich glucose (YPGA) and glycerol (N3) media of serial dilutions of *WT*, *Δatp9*, and *Δatp9* transformed with yAtp9-Nuc. B) Total cellular (*T*), mitochondrial (*M*) and post-mitochondrial supernatant (*C*) protein extracts were prepared from *WT* and *Δatp9*+yAtp9-Nuc strains. Samples were separated via SDS-PAGE and probed with antibodies against yeast Atp9p and the cytosolic protein Pgk1p (phosphoglycerate kinase). C) Western blot of total proteins prepared from *WT* and *Δatp9* yeast transformed with yAtp9-Nuc with or without the *YME1* gene (*Δyme1*) reveals that the yAtp9-Nuc protein is degraded by the i-AAA protease (an oligomer of Yme1p).

In order to express the yeast *ATP9* gene from nuclear DNA, we synthesized a version codon-optimized for nuclear expression in yeast and fused to a mitochondrial targeting sequence (MTS) from the subunit 9 precursor encoded by the nuclear gene *PaAtp9-7* in *P. anserina* (named yAtp9-Nuc, Figure S2 and S3A). In this filamentous fungus, subunit 9 is produced by two different nuclear genes, *PaAtp9-7* and *PaAtp9-5*, that are differentially expressed during its life cycle [2]. When tested on a recoded yeast *ATP8* gene, which can be functionally expressed from nuclear DNA [17], we found the *PaAtp9-7* MTS to be effective in yeast, as evidenced by a complete rescue of a *Δatp8* strain (not shown). This experiment supports our design of the yAtp9-Nuc and its MTS (detailed in Figure S2), which we based on subunit 9 in *Neurospora crassa* [22]. Nonetheless, upon transformation with yAtp9-Nuc, *Δatp9* yeast did not grow on respiratory substrates even after more than 20 days (Figure 2A). Using antibodies against yeast Atp9p, only a protein with the size of the unprocessed yAtp9-Nuc was detected in very small amounts and exclusively in mitochondria (Figure 2B). This indicates that yAtp9-Nuc is targeted to mitochondria, but cannot cross the mitochondrial inner membrane. Since the levels of this protein are so low, we hypothesized that it had been degraded, most likely by the i-AAA protease (a homo-oligomer of Yme1p) that preferentially degrades membrane proteins and whose catalytic site faces the intermembrane space [23]. We confirmed the i-AAA-mediated degradation of yAtp9-Nuc by expressing the latter protein in a *Δyme1* strain. In this strain, the unprocessed protein was far more abundant, while the mature form remained almost absent. These observations verify the defective *in vivo* transport of yAtp9-Nuc across the inner membrane as well as its rapid degradation in the intermembrane space (Figure 2C).

### Nuclear Expression of *P. anserina Atp9* Genes Complements *Δatp9* Yeast

**Figure 3:**
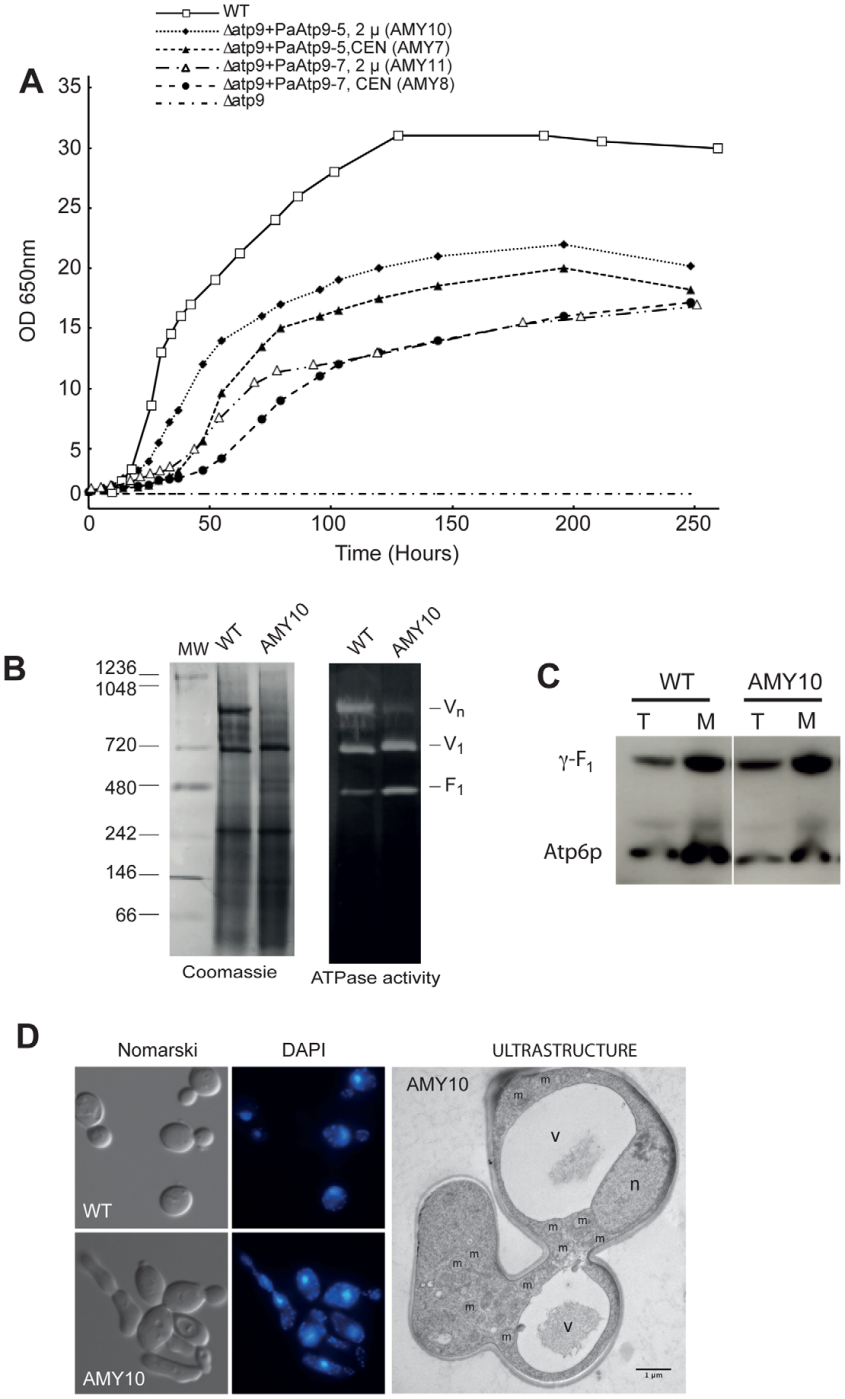
The *P. anserina Atp9* genes restore respiratory competence in *Δatp9* yeast. The *Δatp9* strain was transformed with CEN or 2 µ plasmids harbouring *PaAtp9-7* or *PaAtp9-5*, yielding strains AMY8 (*Δatp9+PaAtp9-7, CEN*), AMY11 (*Δatp9+PaAtp9-7*, 2 µ), AMY7 (*Δatp9+PaAtp9-5*, CEN), and AMY10 (*Δatp9+PaAtp9-5*, 2 µ). A) Growth curves of all strains in rich glycerol/ethanol medium at 28°C (subsequent panels use the same growth conditions). B) ATP synthase levels in *WT* and AMY10 revealed by separation of isolated mitochondria by BN-PAGE. The ATP synthase complexes (*V_1_*, monomer; *V_n_*, oligomers) and free F_1_ were revealed in-gel by their ATPase activity (right). C) Total cellular (*T*) and mitochondrial (*M*) protein extracts were prepared from *WT*, AMY7 and AMY10 grown in YPEG. Samples (20 µg) were separated by SDS-PAGE and probed with antibodies against yeast Atp6p and γ-F_1_. D) Differential interference contrast microscopy (left, ‘Nomarski’) and DAPI staining/fluorescence microscopy (middle) of *WT* and AMY10 cells. Right (‘Ultrastructure’) are electron micrographs of AMY10 cells (80 nm-thin sections). *V*, vacuole; *n*, nucleus; *m*, mitochondria.

We next tested allotopic expression of subunit 9 in yeast using the naturally nuclear *Atp9* genes from *P. anserina* (*PaAtp9-7* and *PaAtp9-5*). The subunit 9 proteins encoded by these genes display 70% amino acid sequence identity with yeast Atp9p (Figure S2). We designed synthetic *PaAtp9-5 and PaAtp9-7* genes codon-optimized for high expression levels in yeast under control of the Tet-off (doxycyclin-repressible) promoter and cloned into both centromeric (pCM189, CEN) and multicopy (pCM190, 2 µ) plasmids (see Figure S3B–C for nucleotide sequences). Upon transformation with each of these plasmids (pAM17 (*PaAtp9-7*, CEN), pAM20 (*PaAtp9-7*, 2 µ), pAM16 (*PaAtp9-5*, CEN) and pAM19 (*PaAtp9-5*, 2 µ)), the *Δatp9* strain regained respiratory capacity, although not to the extent of the *WT* (Figure 3A). The *PaAtp9-5* gene enabled better growth than *PaAtp9-7*, and for both genes, the multicopy plasmid was more effective than the centromeric one. Consistent with the growth measurements, *PaAtp9-5* confers more effective mitochondrial functions related to oxidative phosphorylation than *PaAtp9-7*, as evidenced by the analysis of strains AMY10 (*Δatp9*+*PaAtp9-5*, 2 µ) and AMY11 (*Δatp9*+*PaAtp9-7*, 2 µ) (Table 2). The oxygen consumption rates in AMY10 and AMY11 mitochondria (at state 3 with NADH as an electron donor) were respectively estimated at 80% and 40% of the *WT* rate, and the rates of ATP synthesis at 50% and 25%. This decrease in oxidative phosphorylation capacity is mainly due to reduced amounts of respiratory complexes and assembled ATP synthase (Figure 3B). Oligomeric forms of the ATP synthase were much less abundant than the monomeric form in AMY10 and AMY11 (shown for AMY10 in Figure 3B).

**Table 2:** Respiratory and ATP hydrolysis/synthesis activities of mitochondria.

Strain Genotype (Name)	Growth medium	Oxygen consumption (nAtO_2_/min/mg)		ATP synthesis (nmol ATP/min/mg)	ATP hydrolysis (nmol Pi/min/mg)		
		NADH+ADP	NADH+CCCP		− Oligo	+ Oligo	% Inhibition
*WT* (MR6)	Glycerol+Ethanol	653±58	1261±166	769±55	4159±316	549±60	87
*Δatp9*+*PaAtp9-5* (2 µ) (AMY10)	Glycerol+Ethanol	516±46	1081±17	377±50	3717±866	2783±518	33
*Δatp9*+*PaAtp9-7* (2 µ) (AMY11)	Glycerol+Ethanol	270±38	522±89	186±57	ND	ND	ND
*WT* (MR6)	Galactose	613±12	1081±107	637±18	4474±222	665±108	88
*Δatp9* (RKY26)	Galactose	31±4	44±12	<10	3086±164	2700±180	14

Mitochondria were isolated from yeast cells grown at 28°C in rich glycerol+ethanol (YPEG) or rich galactose (YPGALA), as indicated. All cultures contained less than 5% ρ^−^/ρ^0^ cells, except that of *Δatp9* where about 50% ρ^−^/ρ^0^ cells were scored. Additions were 0.15 mg/ml proteins, 4 mM NADH, 150 mM ADP, 4 mM CCCP, and 3 µg/ml oligomycin (*Oligo*). The values reported are averages of triplicate assays ± standard deviation. Respiratory and ATP synthesis activities were measured on freshly isolated, osmotically-protected mitochondria buffered at pH 6.8. For the ATPase assays, mitochondria kept at −80°C were thawed and the reaction was performed in absence of osmotic protection at pH 8.4. *ND*, not determined.

The reduced ATP synthase levels in the strains expressing the *P. anserina* genes could be due to poor expression, transport, processing or assembly of subunit 9; our evidence indicates the latter is responsible. In a previous study [24], we found that the centromeric pCM189 plasmid enabled sufficient expression of yeast ATP synthase subunit δ, present at one copy per ATP synthase complex. The expression level of the *PaAtp9* genes from the multicopy plasmid pCM190 should thus be sufficient to produce the ten required copies of Atp9p per ATP synthase complex. Unfortunately, our yeast Atp9p antibodies did not recognize the *P. anserina* proteins (see Figure S2), and attempts to raise antibodies directly against *PaAtp9-5* and *PaAtp9-7* were unsuccessful. We constructed HA-tagged version of these proteins, but they failed to complement *Δatp9* yeast. We were therefore unable to directly assess the levels of processed, unprocessed, and unassembled subunit 9 by western blot. Yet several lines of evidence indicate that F_O_ assembly was less effective with the *PaAtp9* proteins. One is the reduced sensitivity of mitochondrial ATPase activity to oligomycin, a specific F_O_ inhibitor, in these strains (<40% *vs.* 80% for *WT*, Table 2). As Atp9p is a constituent of the F_O_ and the F_1_ can assemble independently of F_O_ [25], inefficient expression of the *PaAtp9-7* and *PaAtp9-5* proteins would cause free F_1_ to accumulate, reducing the sensitivity of mitochondrial ATPase activity to oligomycin. A lower yield of F_O_ is additionally supported by higher levels of free F_1_ in the mitochondria of the modified yeast strains than in *WT* (BN-PAGE for AMY10 in Figure 3B) and by diminished Atp6p levels (30–50%), while γ -F_1_ protein levels were unaffected in both total and mitochondrial protein extracts (Figure 3C). As Atp6p is rapidly degraded when it cannot be incorporated into ATP synthase [26], the steady state level of this protein is a good indicator of the amount of assembled subunit 9-ring. The subunit 6 deficiency correlates with the respective reductions in the rate of ATP synthesis in the yeast strains expressing the *PaAtp9* genes. On the other hand, the catalytic efficiency of ATP synthesis (number of ATP molecules synthesized per electron transferred to oxygen) was comparable to that of *WT* yeast (Table 2), meaning that once in the ATP synthase complex the *PaAtp9-5* and *PaAtp9-7* proteins properly interact with other F_O_ components and the F_1_ sector. These results show that the reduced sensitivity of mitochondrial ATPase activity to oligomycin and the higher levels of free F_1_ in the modified yeast strains are most likely due to an imbalanced production of F_O_ and F_1_, which is a result of less efficient Fo assembly in these strains.

ATP synthase is known to be far from limiting for respiratory growth in yeast, since a deficit in its activity of at least 80% is required before a growth defect can be observed [27], [28]. Thus, it was expected that with respective ATP synthesis levels of 50% and 25%, AMY10 and AMY11 would grow far better than we observed. This discrepancy is likely because expressing the *PaAtp9-5* and *PaAtp9-7* genes causes deleterious side effects in yeast. For example, the modified yeast strains displayed aberrant, pseudohyphal cell morphology when grown in glycerol and ethanol medium (shown for AMY10, Figure 3C). It is not clear how this phenotype arises, but it very likely impedes respiratory growth of *Δatp9* yeast expressing the *PaAtp9* genes.

### Reducing the Hydrophobicity of the First Transmembrane Segment of Subunit 9 Is Required for Its Nuclear Expression

**Figure 4:**
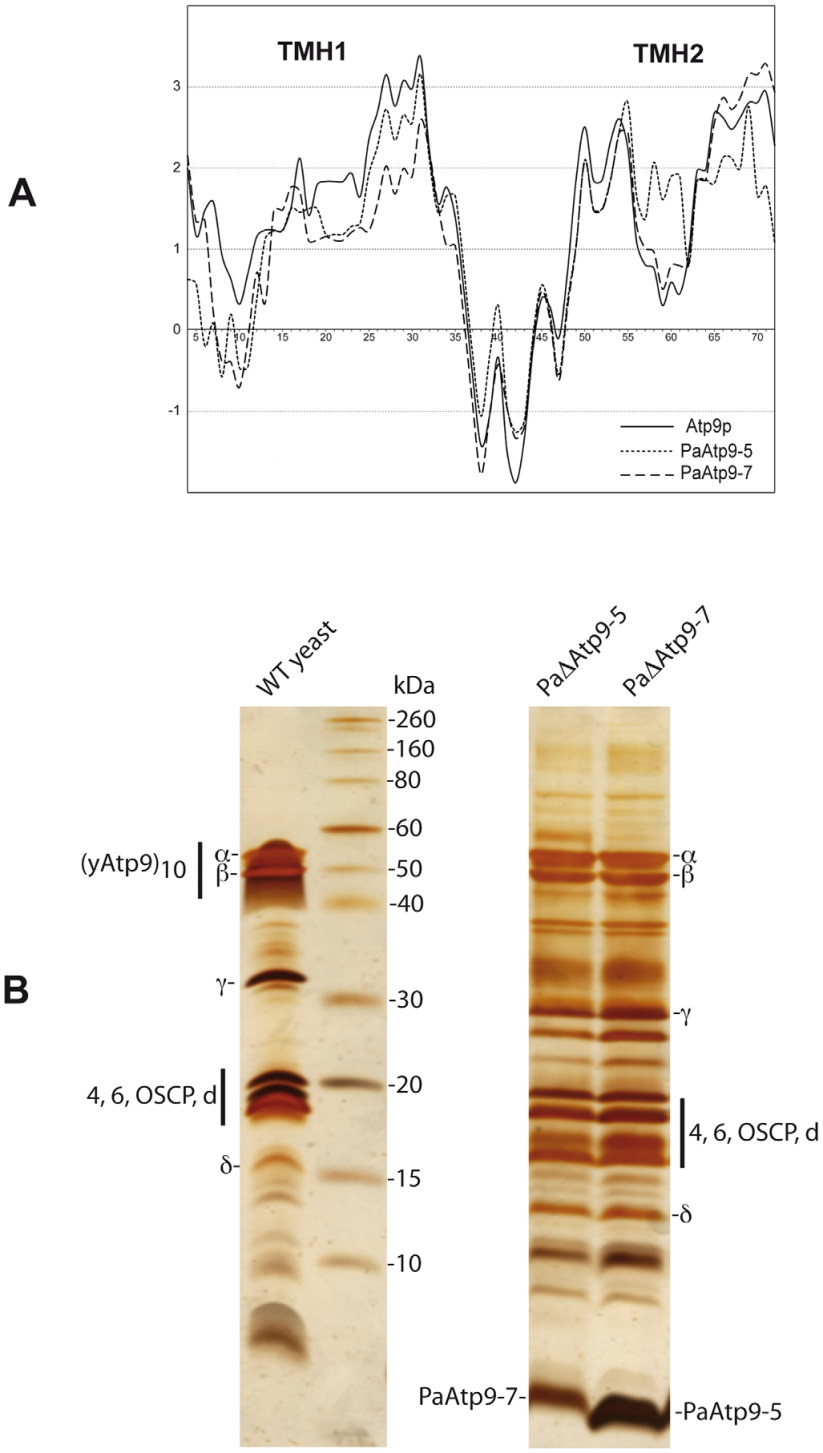
The *P. anserina Atp9* proteins are less hydrophobic than yeast Atp9p. A) Hydropathy profiles of the *PaAtp9-7* and *PaAtp9-5* proteins and yeast Atp9p, generated according to [54] with a window size of 13. B) *P. anserina* strains expressing exclusively either *PaAtp9-7* (PaΔAtp9-5) or *PaAtp9-5* (PaΔAtp9-7) were constructed and ATP synthase was enriched from their mitochondrial extracts, separated by SDS-PAGE and silver-stained along with *WT* yeast ATP synthase. Positions of some ATP synthase subunits are indicated. The *PaAtp9*-5 protein is stained much more strongly than the *PaAtp9-7* protein, which may be due to the differences in their amino acid sequences.

The results described above are evidence that structural modification of subunit 9 is required for functional expression of this protein from nuclear DNA. A striking difference between the *PaAtp9* proteins and yeast Atp9p is reduced hydrophobicity in the first transmembrane segment of the former, as indicated by hydropathy plots (Figure 4A). Additional evidence of reduced hydrophobicity results from comparing the resistance of the subunit 9-ring to detergents in *P. anserina* and yeast. To make this comparison, we enriched mitochondrial extracts for ATP synthase from engineered *P. anserina* strains expressing only *PaAtp9-7* or *PaAtp9-5* (details in Text S1). On a denaturing SDS gel, the *PaAtp9* proteins were both detected only as monomers, whereas yeast Atp9p was mainly present as oligomeric rings (Figure 4B). These results indicate that the inter-subunit interactions responsible for maintaining the subunit 9-rings are significantly less hydrophobic in *P. anserina* than in yeast.

**Figure 5:**
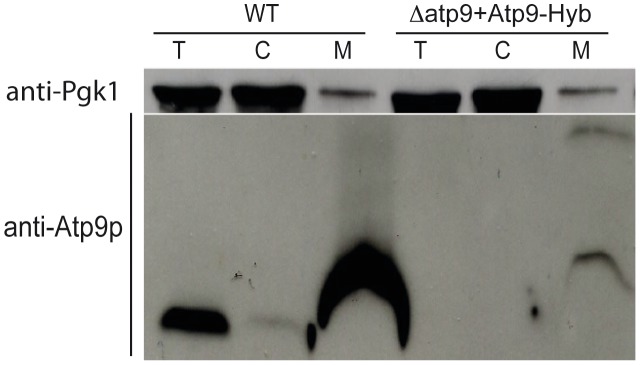
Reducing the hydrophobicity of the first transmembrane segment of yeast Atp9p improves its import into mitochondria. The *Δatp9* strain was transformed with a hybrid Atp9 gene (Atp9-Hyb) encoding the mitochondrial targeting sequence (MTS) and first transmembrane segment (TMH1) of the *PaAtp9-7* protein, followed by the connecting loop and second transmembrane segment (TMH2) of yeast Atp9p (see Figure 2A for amino acid sequence and Figure S3E for nucleotide sequence). Total cellular (*T*), mitochondrial (*M*) and post-mitochondrial supernatant (*C*) protein extracts were prepared from *WT* and *Δatp9*+Atp9-Hyb strains. Samples were separated by SDS-PAGE and probed with antibodies against yeast Atp9p and the cytosolic protein Pgk1p (phosphoglycerate kinase).

To test whether hydrophobicity of the first transmembrane segment of subunit 9 affects expression of this protein from nuclear DNA, we designed a hybrid gene (Atp9-Hyb) encoding the MTS and first transmembrane segment of the *PaAtp9-7* protein, followed by the connecting loop and second transmembrane segment of yeast Atp9p (see Figure S2 and S3D for protein and nucleotide sequences). Although *Δatp9* yeast expressing Atp9-Hyb grew extremely slowly on glycerol (not shown), some processed hybrid protein was detected in mitochondria (Figure 5) whereas, as shown above (Figure 2B), only trace amounts of unprocessed protein were detected in *Δatp9* yeast expressing a nuclear version of the endogenous yeast *ATP9* gene. Taken together, these data indicate that the high hydrophobicity of the first transmembrane segment of yeast Atp9p prevents its translocation across the mitochondrial membranes from the cytosol.

### Transcription Profiling Reveals Regulatory Consequences of *ATP9* Relocation

**Figure 6:**
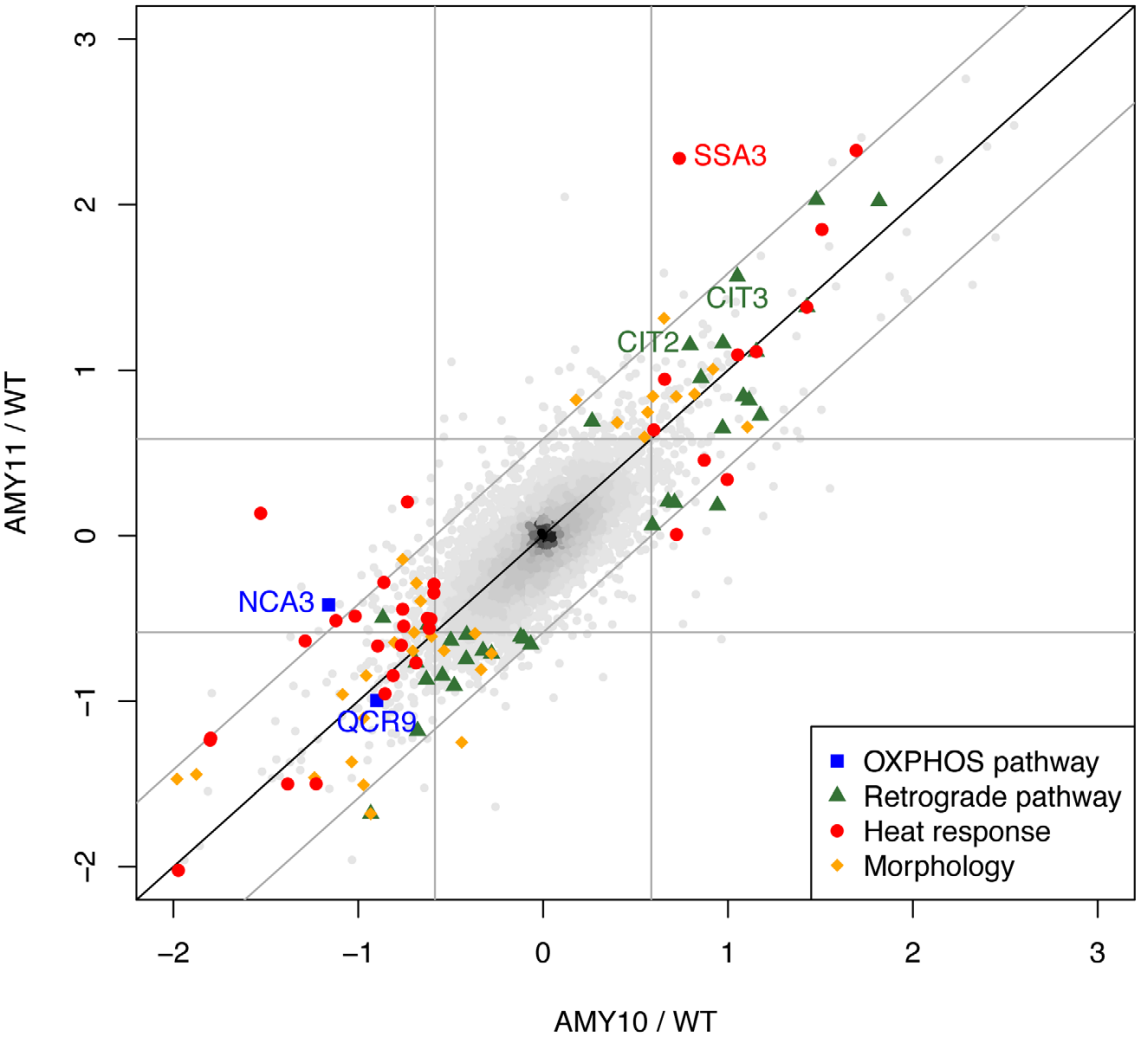
Transcriptome profiles of yeast strains expressing *P. anserina Atp9* genes indicate functional OXPHOS and regulatory responses to the nuclear relocation of *ATP9*. For all genes in the yeast genome, the expression levels in AMY11 (expressing *PaAtp9-7*) are plotted against those of AMY10 *(PaAtp9-5)*, both displayed as log_2_ ratios to *WT* expression levels; differentially expressed genes in the main functionally relevant categories are indicated by colours. The square formed by the grey lines delineates the boundaries of statistically significant expression differences (see *Text S1*) between either strain and the *WT*; genes beyond the diagonal grey lines are differentially expressed between AMY10 and AMY11. For clarity, genes in the categories listed are only indicated if they were differentially expressed relative to *WT* in at least one strain. Categories were defined as follows: OXPHOS pathway - subunits (1/35 differentially expressed) and biogenesis factors (1/42); Retrograde pathway – transcriptional targets of the factors Gcn4p (29/126) and Rtg3p (6/31), plus *CIT2* and *CIT3*; Heat response - Gene Ontology (GO)-annotated “response to heat” genes (28/199); Morphology - Phd1p targets (23/81), plus GO “cell-cell adhesion” (2/4) and “cytokinesis, completion of separation” genes (6/11). All categories except OXPHOS were significantly enriched among differentially expressed genes (according to Fisher's exact test with multiple hypothesis testing correction, or to Model Gene Set Analysis (MGSA); see *Text S1*).

To investigate the effects of expressing the *P. anserina ATP9* genes in *Δatp9* yeast at a cellular level, we carried out whole-genome transcription profiling using high-resolution tiling microarrays (data browsable at http://steinmetzlab.embl.de/atp9/; results of analysis in Dataset S1; raw data in ArrayExpress database; functional analysis in Figure 6; intercorrelation of samples in Figure S4). Notably, no functions related to oxidative phosphorylation are enriched in genes differentially expressed between any of the relocation strains and *WT* (Figure 6), indicating that the complementation is mostly sufficient for the function of the respiratory chain. The only genes directly involved in OXPHOS whose expression changes when *ATP9* is relocated are *QCR9* and *NCA3*. Since the latter regulates translation of *ATP6* and *ATP8* [29] and the former may play a role in cytochrome *bc*_1_ biogenesis [30], the downregulation of these genes supports the biochemical indications of an overall decrease in respiratory chain biogenesis and assembly. Some signals of the retrograde response, a typical sign of respiratory deficiency, also suggest that the complementation is incomplete (Figure 6). At the cellular level, we also see evidence of the response to overproduction of a new hydrophobic protein in the cytoplasm via enrichment of the Gene Ontology term “response to temperature stimulus” (MGSA, posterior probability >0.85), which entails modulation of protein folding. Notably, the gene *SSA3* is among the most strongly upregulated in the relocation strains, which encodes an HSP70 chaperone protein involved in co-translational protein-membrane targeting and whose overexpression protects a yeast model of Parkinson's disease from alpha-synuclein toxicity [31]; it is therefore possible that this protein helps to prevent toxic aggregation of the *PaAtp9* proteins. The morphological abnormalities of the relocation strains are clarified by the transcription profiles, in particular by functional enrichments like “cell separation during cytokinesis” among downregulated genes (P = 0.003) and “cell-cell adhesion” among upregulated genes (P = 0.008) as well as enrichments for targets of the transcription factor *PHD1* (MGSA, posterior probability >0.95), a master regulator of pseudohyphal growth (Figure 6). Taken together, the transcriptome data corroborate the biochemical observations while providing more specific insights into the cellular outcome of relocating *ATP9* to the nucleus in yeast, particularly in terms of the difficulties in folding the foreign protein.

## Discussion

Our study demonstrates that allotopic expression of the yeast mitochondrial *ATP9* gene is possible, provided that sufficient modifications to the protein structure are made. Despite previous reports that yeast Atp9p can be imported by isolated wild-type mitochondria [18], we found that expressing the yeast *ATP9* gene from nuclear DNA (yAtp9-Nuc construct, see Figure 2) does not restore *in vivo* respiratory function in a *Δatp9* strain. Although the precursor protein is correctly targeted to mitochondria, it cannot cross the mitochondrial inner membrane and is degraded in the intermembrane space by the i-AAA protease (Figure 2).

**Figure 7:**
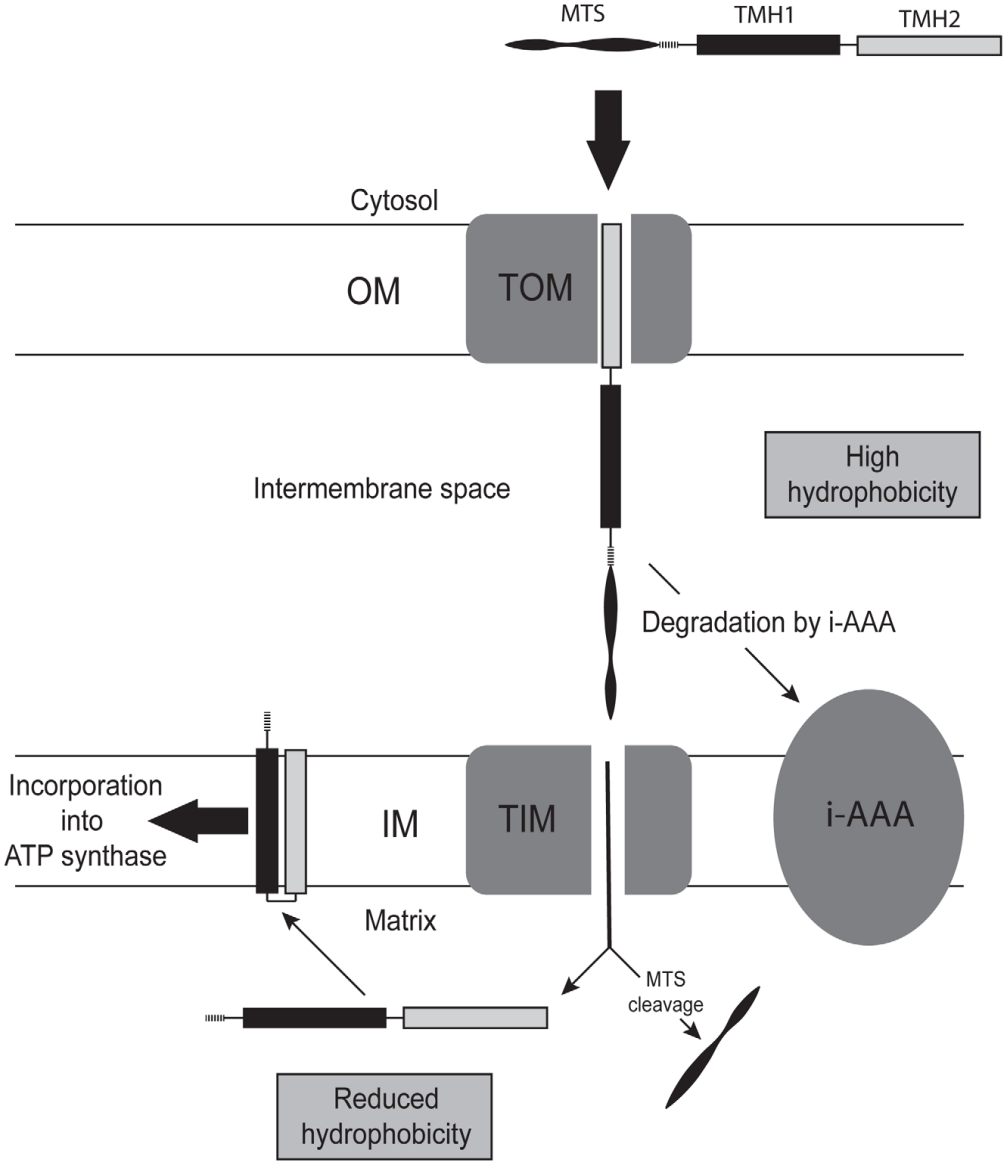
Model of how a reduction in the hydrophobicity of subunit 9 permits its functional expression from nuclear DNA. When the hydrophobicity of subunit 9 is too high, the protein cannot cross the inner mitochondrial membrane (IM) and is degraded in the intermembrane space by the i-AAA protease. With reduced hydrophobicity, subunit 9 can cross the IM and is processed by the matrix processing peptidase (MPP), properly inserted into the IM, and assembled into ATP synthase (see text for details). OM, outer mitochondrial membrane; MTS, mitochondrial targeting sequence, TMH, transmembrane segment; TOM, translocase of the OM; TIM, translocase of the IM.

We achieve allotopic expression of ATP synthase subunit 9 using the nuclear *Atp9* genes of *P. anserina* (*PaAtp9-5* and *PaAtp9-7*). Remarkably, despite only 70% sequence identity between these proteins and yeast Atp9p, the production of functional ATP synthase was significantly restored (30–40%) in *Δatp9* yeast and the ‘hybrid’ enzymes displayed high catalytic efficiency. This indicates that the high hydrophobicity of yeast Atp9p relative to the *P. anserina* proteins impedes expression of the former from nuclear DNA. This constraint is also evident in the case of ATP synthase subunit 6, which contains five membrane-spanning segments. In the few species where this protein is nuclear-encoded, the first three transmembrane segments of subunit 6 are significantly less hydrophobic than the ones encoded by mitochondrial genomes [32]. Several of our results indicate that reducing the hydrophobicity of subunit 9 is also required for nuclear relocation. Hydropathy plots (Figure 4A) indicate significantly lower hydrophobicity in the first transmembrane segment of the *P. anserina Atp9* proteins; in addition, the *P. anserina* subunit 9-rings can be dissociated much more easily with detergents than the yeast Atp9p-ring (Figure 4B). Finally, when substituting the first transmembrane segment of yeast Atp9p with that of the *PaAtp9-7* protein (Atp9-Hyb construct), mature protein was detected in mitochondria (Figure 5), which was not observed with nuclear expression of the endogenous yeast *ATP9* gene (Figure2B). From these results, we conclude that reducing the hydrophobicity of the first transmembrane segment of subunit 9 is necessary for this protein to cross the mitochondrial inner membrane, after which it can be inserted into the membrane and correctly folded for incorporation into ATP synthase (Figure 7). These results clearly demonstrate the importance of protein structure and hydrophobicity in transferring mitochondrial genes to the nucleus.

Additional adaptations beyond protein structure are required to optimize allotopic expression of subunit 9; in particular, transcriptional evidence of the retrograde response in the relocation strains indicates that their mitochondria are not functioning optimally (Figure 6). Indeed, although the ATP synthase in *Δatp9* yeast expressing the *P. anserina Atp9* genes is catalytically efficient, the yield in complex assembly is quite low compared to wild-type yeast (30–40%). One possible reason is that the *P. anserina* proteins are more exposed to proteases than the native yeast Atp9p, the latter being inserted into the membrane during its own synthesis in mitochondria [26]. The upregulation of *SSA3* in the relocation strains, a gene that has been implicated in cotranslational import [31], may similarly have facilitated the transport of the *PaAtp9* proteins across the outer mitochondrial membrane; this, however, would not protect them from mitochondrial proteases. In addition, yeast ATP synthase assembly is a remarkably evolved and sophisticated system: multiple factors execute highly specific functions ranging from synthesis to oligomerization of the individual subunits [25], [26]. The factors that assemble *ATP9* depend on its expression from the mitochondria; it is therefore natural that this process would be less efficient when the protein is coming from an unexpected direction. Decreased biogenesis of ATP synthase is also supported by the reduced expression of *NCA3*, a gene that regulates expression of Atp6p and Atp8p [29]. Effective nuclear expression of subunit 9 may also require adaptations beyond mitochondrial biogenesis, as suggested by the aberrant cellular morphology of strains expressing the *P. anserina Atp9* genes (Figure 3C). We have therefore undertaken the selection of mutations that improve allotopic expression of subunit 9, a strategy that could bring new insight into the mechanisms involved in organellar gene transfer during the evolution of eukaryotes. Exploration of these mechanisms holds promise for developing therapeutic strategies for human diseases caused by mitochondrial DNA mutations. There is no report thus far with strong functional and biochemical evidence that allotopically expressed proteins are properly incorporated into OXPHOS complexes in human cells [33], [34]. Our yeast-based approach has potential to unravel the general adaptations necessary for expressing mitochondrial proteins from nuclear DNA.

A recent survey of the *ATP9* gene in 26 fungal species has revealed a strong diversity of genomic locations of this gene, especially in the *Pezizomycotina* subphylum which is comprised primarily of filamentous fungi [2]. Phylogenetic data indicate that early in the evolution of this group of fungi, *ATP9* was transferred to the nucleus twice, which was eventually followed by independent mitochondrial gene loss events. As a result, five different distributions of the *ATP9* gene between the nuclear and mitochondrial genomes can be found in filamentous fungi. The complete lack of functional expression of yeast Atp9p from the nucleus and partial complementation by a naturally nuclear version of this protein demonstrate that effective nuclear relocation of the *ATP9* gene requires multifactorial adaptations; it would therefore probably not occur during evolution unless it conferred significant benefits to the organism. This idea is supported by a recent study in *P. anserina* where subunit 9 is produced by two nuclear genes (*PaAtp9-5* and *PaAtp9-7*) and where the ancestral mitochondrial gene copy has been lost [2]. *PaAtp9-5* is strongly expressed in germinating spores, where a high rate of ATP synthase production is necessary. In the sexual reproduction phase, when external nutrients become limited, the fungus ceases vegetative growth and produces spores. This phase does not require large quantities of neosynthesized ATP synthase, and subunit 9 is thus produced almost exclusively at a much lower level by *PaAtp9-7*. Complex regulation of *ATP9* has also been observed in the filamentous fungus *Neurospora crassa*, where one nuclear copy of this gene co-exists with one mitochondrial version; this may be regarded as an intermediate stage of evolution at which the loss of the mitochondrial gene cannot be tolerated [35]. In mammals, as in *P. anserina*, subunit 9 is produced exclusively from multiple nuclear isogenes that are transcriptionally regulated to modulate its production in a cell- and tissue-specific manner [36]. A well-documented case is the pronounced downregulation of subunit 9 in the thermogenic brown adipose tissue (BAT) upon cold acclimation [37]. As a result, the amount of ATP synthase is decreased tenfold in BAT, and due to the concomitant induction of an uncoupling protein (UCP), the proton-motive force of the inner mitochondrial membrane is converted into heat rather than ATP.

It is clearly more effective for cells to respond to changes in their environment by modulating the activity of nuclear genes than mitochondrial genes, which are devoid of gene-specific transcriptional regulation [19], [38]. The *ATP9*-mediated regulation of ATP synthase production in fungi and mammals requires a nuclear location of this gene. Notably, the nuclear relocation of *ATP9* occurred independently in fungi and mammals, and in both kingdoms the regulation of this gene was specialized such that it could modulate ATP synthase production according to certain environmental factors. An intriguing question is why this important regulatory function has been attributed to a gene that is so difficult to relocate to the nucleus. Many of the other structural genes of mitochondrial ATP synthase can be transferred much more easily, such as those encoding the F_1_ subunits that are, almost without exception, nuclear [1], [39]. Subunit 9 may be preferentially used as a regulatory target to modulate production of the enzyme because it can do so safely, without accumulating potentially harmful assembly intermediates. In mammals, it is the only subunit whose expression is transcriptionally regulated; when its expression is diminished, the other ATP synthase subunits that are in excess are eliminated by proteolytic degradation [38]. Due to its extremely hydrophobic nature, it may be safer to control subunit 9 at the level of synthesis rather than by proteolytic degradation, similarly to what has been reported for the Cox1p subunit of complex IV [40], [41].

As described in the introduction, several hypotheses have been proposed to account for the retention of DNA in mitochondria: (i) gene transfer from mtDNA is still underway; (ii) some genes have been confined to the organelle because expressing them from the nucleus would be problematic; and (iii) some genes have been preferentially retained in order to optimize mitochondrial function. In the case of *ATP9*, our study demonstrates that this gene can, in principle, be relocated to the nucleus in yeast; nevertheless, the adaptations required to optimize its nuclear expression are many and complex in nature. Thus, there must be a compelling reason for *ATP9* to become nuclear, such as the requirement for more specialized regulation of ATP synthase activity by complex, multicellular organisms. Otherwise it would remain in the organelle, as in all unicellular organisms including *S. cerevisae* where ATP synthase expression is not subject to any specialized regulation beyond the general glucose-induced repression of respiratory functions [25]. Accordingly, we would like to introduce the hypothesis that variations in the gene content of mitochondria are influenced by not only protein structure, but also the lifestyle of the organism.

## Materials and Methods

### Yeast Strains and Culture Medium

The *S. cerevisiae* strains used and their genotypes are listed in Table 1. For details on growth procedures, see *Text S1*.

### Deletion of the Mitochondrial *ATP9* Gene

We deleted the *ATP9* gene using the *ARG8^m^*-based procedure described by T. Fox and colleagues [19]. For details, see *Text S1*.

### Synthetic *ATP9* Genes

The coding sequences of all synthetic *Atp9* genes used in this study (yAtp9-Nuc, *PaAtp9-5*, *PaAtp9-7*, and Atp9-Hyb) were designed for optimal expression in yeast using the Gene Designer sofware by DNA2.0 (www.dna20.com; corresponding amino-acid sequences in Figure S2; nucleotide sequences in Figure S3). The synthetic genes were first cloned into pJ204 and then into the yeast expression vectors pCM190 and/or pCM189 (from ATTC) using *BamHI* and *PstI* restriction sites at their 5′ and 3′ ends (all plasmids in Table S1).

### Deletion of *YME1* in Strain AMY5

We deleted the *YME1* gene in the AMY5 strain (*Δatp9*+y*Atp9*-Nuc) using a PCR-amplified null allele of this gene (yme1::KanMX4, from Euroscarf). For details, see *Text S1*.

### *In Vivo* Labelling of Mitochondrial Translation Products

Cells were grown overnight in CSM galactose to early exponential phase (10^7^ cells/ml). One ml of cells was harvested by centrifugation and washed with 40 mM potassium phosphate pH 6.0 containing 2% galactose. The cells were suspended in 500 µl of the same buffer and 15 µl of a freshly prepared aqueous solution of cycloheximide (7.5 mg/ml) was added. The cell suspension was incubated at 24°C for 5 min prior to addition of 50 µCi of ^35^S-methionine (100 Ci/mmole, GE Healthcare, Piscataway, NJ). The reaction was terminated after 10 min by adding 500 µl of 20 mM methionine and 75 µl of 1.8 M NaOH, 1 M β-mercaptoethanol and 0.01 M PMSF. An equal volume of 50% TCA was added and the mixture was centrifuged for 5 min at 14,000 rpm. The precipitated proteins were washed once with 0.5 M Tris (free base), twice with water, and finally resuspended in 45 µl of Laemmli buffer [42].

### Respiratory and ATP Synthesis/Hydrolysis Activities of Isolated Mitochondria

Mitochondria were prepared by the enzymatic method as described [43]. For details on measurements of respiratory and ATP synthesis/hydrolysis activities, see *Text S1*.

### Northern and Southern Blot Analyses

Northern blot analyses were performed using total cellular RNA extracted from yeast cells as previously described [44]. The Southern blot analyses were done using mitochondrial DNA isolated as previously described [45]. See *Text S1* for details.

### Miscellaneous Procedures

Proteins were separated by SDS-PAGE as previously described [42]. Blue-native PAGE was carried out according to [46]. Protein amounts were determined by the Lowry procedure [47] in the presence of 5% SDS. Western blot analyses were performed as described in [48]. Polyclonal antibodies raised against yeast ATP synthase (from J. Velours) were used at dilutions of 1∶10,000 for subunit 4; 1∶5,000 for Atp6p; 1∶7,500 for Atp9p; 1∶5,000 for γ-F_1_. Polyclonal antibodies against cytochrome *b* (from T. Langer), Yme1p (from T. Langer) and Arg8p (from T. Fox) were used at dilutions of 1∶2,000, 1∶10,000, and 1∶200 respectively. Monoclonal antibodies against Cox2p, Pgk1 and porin (from Molecular Probes) were used at a dilution of 1∶500. Nitrocellulose membranes were incubated with rabbit peroxidase-labeled antibodies at a 1∶10,000 dilution and revealed with ECL reagent (Amersham Biosciences International). Electron microscopy analyses of yeast were performed as previously described [49].

### Purification of ATP Synthase

Yeast ATP synthase was purified as previously described [50]. A modified version of this protocol was used to purify ATP synthase from the *P. anserina* strains expressing either *PaAtp9-7* or *PaAtp9-5* (see below and *Text S1* for details).

### Construction of *P. anserina* Strains for Purification of ATP Synthase

Two *P. anserina* strains were constructed to facilitate purification of ATP synthase exclusively containing either the subunit 9 encoded by *PaAtp9-7* (strain Δ5) or that encoded by *PaAtp9-5* (strain Δ7). In both strains the C-terminus of ATP synthase subunit *j* (equivalent of yeast subunit *i*) was fused to a (His)_6_ tag, followed by a nourseothricin resistance gene cassette (*nat1*) 540 nt dowstream from the potential polyA site of the *j* subunit gene. For details, see *Text S1*.

### Transcription Profiling

For each analyzed strain, two biological replicates were cultured (see *Text S1* for growth conditions), harvested, and hybridized to whole-genome tiling arrays as previously described, except that polyA RNA was isolated using the QIAGEN Oligotex mRNA isolation kit prior to cDNA synthesis [30]. Raw tiling array data were processed as described in *Text S1*. Data can be browsed at http://steinmetzlab.embl.de/atp9, normalized data is provided in Dataset S1, and raw data is available from the ArrayExpress database (accession: E-MTAB-1115).

## Supporting Information

Dataset S1 **Normalized gene expression levels from transcription profiles.** Gene expression values per ORF across the entire yeast genome for *WT* and strains expressing the *PaAtp9* genes grown in rich ethanol/glycerol media, summarized following normalization by the median intensity of all probes per ORF. Rows correspond to ORFs, columns to strains (MR6, AMY7, AMY8, AMY10, and AMY11), including both the expression levels (columns 2–6) and false discovery rates (columns 7–10) indicating whether the expression level is significantly different from the WT (the cutoff used in this study was FDR<0.1). Values are NA if there were insufficient probes to estimate the intensity levels, and 0 if the probe intensity levels were deemed below background (see *Text S1*).
(ZIP)

Figure S1 **Construction of** *Δatp9* **yeast and additional phenotypic properties.** A) Schematic of the wild-type (*WT*) and deleted *atp9::ARG8^m^* loci (*Δatp9*). The broken arrow indicates the main transcription initiation site of the polycistronic unit which contains *ATP9*. Positions and sizes of restriction fragments (*a–e*) used to confirm replacement of *ATP9* with *ARG8^m^* by Southern blot (B) are indicated. B) Southern blot analysis. Mitochondrial DNA was extracted from *WT*, a ρ^0^ derivative of *WT*, and *Δatp9*, and hybridized to ^32^P-labeled *ATP9* and *ARG8^m^* probes. Positions and sizes of the bound DNA fragments (*a–e*) are indicated in A). Th*e ATP9* probe is the PCR fragment amplified with the primers 9-4 (5′- TATGCAATTAGTATTAGCAGC) and 9–67 (5′- GAATGTTATTAATTTAATCAAATGAG), and the *ARG8^m^* probe is the entire *atp9::ARG8^m^* cassette amplified with the primers OligoproATP9 and OligoTermATP9 (primer sequences in *Text S1*). C) Northern blot analysis of mitochondrial transcripts: Total RNA from *WT* and *Δatp9* was hybridized to radiolabelled probes specific to *ATP9*, *ARG8^m^*, *ATP6,8* (long (*L*) and short (*S*)), *COX1* and *15S rRNA*. D) Western blots of mitochondrial proteins. E) BN-PAGE of respiratory chain complexes III and IV: proteins from *WT* and *Δatp9* mitochondria were extracted with digitonin and revealed by western blotting with antibodies against Cox1p or cytochrome *b*. F) Complementation of *Δatp9* by a synthetic ρ^−^ strain carrying only *ATP9* (ρ^−^ ATP9, strain MB2). The two strains were crossed ‘drop on drop’ and incubated for two days on rich glucose with non-mated control strains on the same plate. The plate was then replicated on rich glycerol and photographed after 5 days of incubation.
(PNG)

Figure S2 **Protein sequences and alignments.** Amino acid sequences and alignments of the proteins encoded by the *Atp9* genes from *P. anserina* (PaAtp9-7 and PaAtp9-5), *N. crassa* (NcAtp9), *S. cerevisiae* (Atp9p), a nuclear version of the *S. cerevisiae* protein (yAtp9-Nuc, see Figure S3A for nucleotide sequence), and a chimeric protein (Atp9-Hyb, Figure S3E for nucleotide sequence) composed of the mitochondrial targeting sequence (MTS) and first transmembrane segment (TMH1) of the *PaAtp9-7* protein by followed by the TMH2 segment of the yeast protein. The underlined amino acids in Atp9p correspond to the peptide used to raise antibodies against the yeast protein (this sequence is not conserved in *P. anserina*, which is why the yeast antibody is ineffective for the *PaAtp9* proteins). The arrowhead points to the site of cleavage of the MTS by mitochondrial processing peptidase (MPP) in *N. crassa* [22]. Based on the *N. crassa* cleavage site, it is likely that *PaAtp9-7* is cleaved after the Glycine 64 residue. Mature *PaAtp9-7* is thus predicted to contain a sequence of six residues (VVAETA) at the N-terminus that has no counterpart in yeast Atp9p. We have nonetheless maintained this sequence as we found that it did not hinder allotopic expression of *ATP8*. Protein lengths are indicated on the right.
(PNG)

Figure S3 **Construction of** *P. anserina ATP9* **gene cassettes.** A) Sequence of *PaAtp9-7* cDNA cloned into pFL61 (pMB6C). When this plasmid was transformed into the *Δatp9* strain RKY26, the resulting strain did not grow on glycerol media, but accumulated revertants following several rounds of selection. We isolated a revertant (strain MBE2) and sequenced the plasmid insert, revealing a precise deletion of the 5′ UTR (boxed sequence). We thus used synthetic genes optimized for yeast expression in subsequent experiments. B) Sequence of a synthetic version of *PaAtp9-5* optimized for expression in yeast, cloned into pCM189 (pAM16) or pCM190 (pAM19). C) Sequence of a synthetic version of *PaAtp9-7* optimized for expression in yeast, cloned into pCM189 (pAM17) or pCM190 (pAM20). D) Sequence of the hybrid/chimeric gene (Atp9-Hyb) encoding the MTS and first transmembrane segment of *PaAtp9-7*, followed by the connecting loop and second transmembrane segment of the protein encoded by yeast *ATP9* (pAM12). Capitalized bases indicate coding sequences. Restriction sites used for cloning are underlined.
(PDF)

Figure S4 **Correlation matrix demonstrating gene expression similarities among** *ATP9* **relocation strains.** Clustering reveals that greater gene expression differences in the relocation strains are induced by gene (*PaAtp9-5 vs. PaAtp9-7*) than by plasmid type (centromeric *vs.* multicopy), and that the biological replicates for each strain (indicated by numbers) display strong reproducibility. Samples were clustered hierarchically using the Euclidean distance between the pairwise Pearson correlation coefficients, which were computed between vectors of all per-gene normalized intensities per sample. Strains were grown in rich ethanol/glycerol media.
(PDF)

Table S1 **Plasmids used in this study.**
(DOCX)

Text S1 **Supplementary methods.**
(DOC)
