# Supplementary figures and images for: Experimental Relocation of the Mitochondrial ATP9 Gene to the Nucleus Reveals Forces Underlying Mitochondrial Genome Evolution
Source: PLoS Genet. 2012 Aug 16;8(8):e1002876. doi: 10.1371/journal.pgen.1002876 (PMC3420929; doi:10.1371/journal.pgen.1002876)

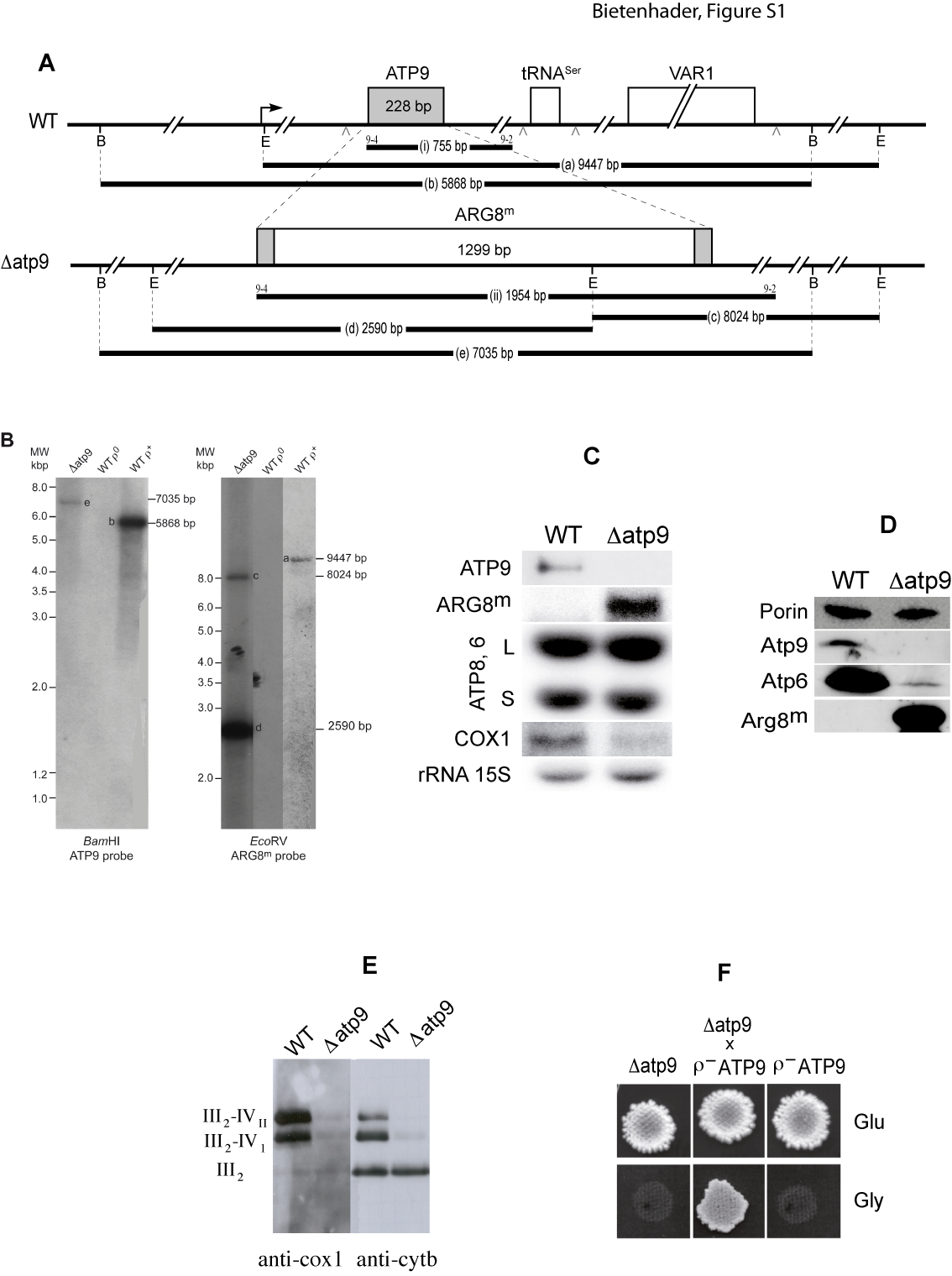

Supplement: Figure S1 — Construction of Δatp9 yeast and additional phenotypic properties. A) Schematic of the wild-type (WT) and deleted atp9::ARG8m loci (Δatp9). The broken arrow indicates the main transcription initiation site of the polycistronic unit which contains ATP9. Positions and sizes of restriction fragments (a–e) used to confirm replacement of ATP9 with ARG8m by Southern blot (B) are indicated. B) Southern blot analysis. Mitochondrial DNA was extracted from WT, a ρ0 derivative of WT, and Δatp9, and hybridized to 32P-labeled ATP9 and ARG8m probes. Positions and sizes of the bound DNA fragments (a–e) are indicated in A). The ATP9 probe is the PCR fragment amplified with the primers 9-4 (5′- TATGCAATTAGTATTAGCAGC) and 9–67 (5′- GAATGTTATTAATTTAATCAAATGAG), and the ARG8m probe is the entire atp9::ARG8m cassette amplified with the primers OligoproATP9 and OligoTermATP9 (primer sequences in Text S1). C) Northern blot analysis of mitochondrial transcripts: Total RNA from WT and Δatp9 was hybridized to radiolabelled probes specific to ATP9, ARG8m, ATP6,8 (long (L) and short (S)), COX1 and 15S rRNA. D) Western blots of mitochondrial proteins. E) BN-PAGE of respiratory chain complexes III and IV: proteins from WT and Δatp9 mitochondria were extracted with digitonin and revealed by western blotting with antibodies against Cox1p or cytochrome b. F) Complementation of Δatp9 by a synthetic ρ− strain carrying only ATP9 (ρ− ATP9, strain MB2). The two strains were crossed ‘drop on drop’ and incubated for two days on rich glucose with non-mated control strains on the same plate. The plate was then replicated on rich glycerol and photographed after 5 days of incubation. (PNG) [file pgen.1002876.s002.png]

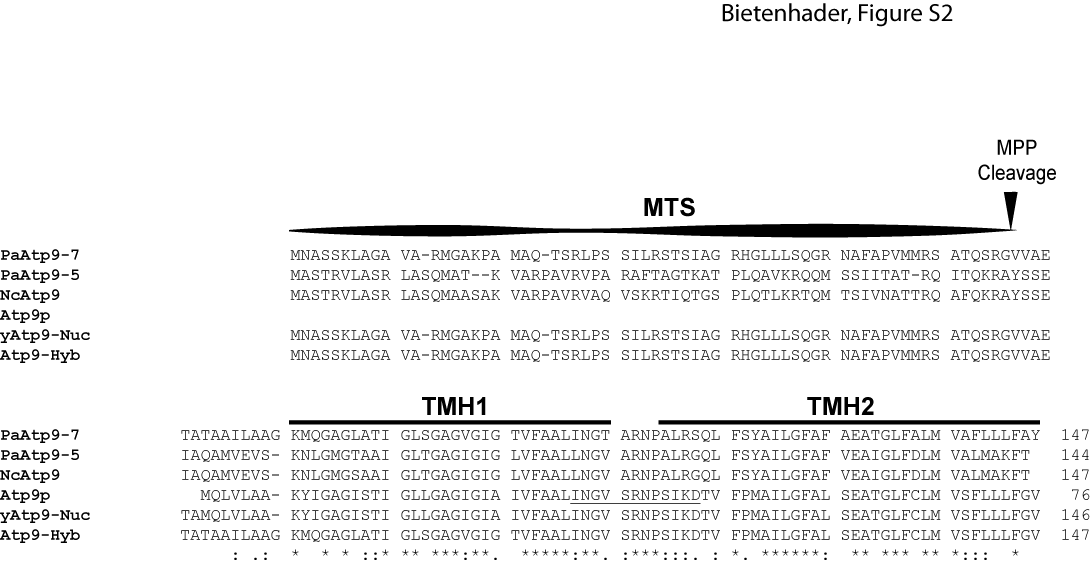

Supplement: Figure S2 — Protein sequences and alignments. Amino acid sequences and alignments of the proteins encoded by the Atp9 genes from P. anserina (PaAtp9-7 and PaAtp9-5), N. crassa (NcAtp9), S. cerevisiae (Atp9p), a nuclear version of the S. cerevisiae protein (yAtp9-Nuc, see Figure S3A for nucleotide sequence), and a chimeric protein (Atp9-Hyb, Figure S3E for nucleotide sequence) composed of the mitochondrial targeting sequence (MTS) and first transmembrane segment (TMH1) of the PaAtp9-7 protein by followed by the TMH2 segment of the yeast protein. The underlined amino acids in Atp9p correspond to the peptide used to raise antibodies against the yeast protein (this sequence is not conserved in P. anserina, which is why the yeast antibody is ineffective for the PaAtp9 proteins). The arrowhead points to the site of cleavage of the MTS by mitochondrial processing peptidase (MPP) in N. crassa [22]. Based on the N. crassa cleavage site, it is likely that PaAtp9-7 is cleaved after the Glycine 64 residue. Mature PaAtp9-7 is thus predicted to contain a sequence of six residues (VVAETA) at the N-terminus that has no counterpart in yeast Atp9p. We have nonetheless maintained this sequence as we found that it did not hinder allotopic expression of ATP8. Protein lengths are indicated on the right. (PNG) [file pgen.1002876.s003.png]

Color Key

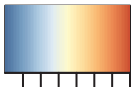

0.97 0.99  
Value

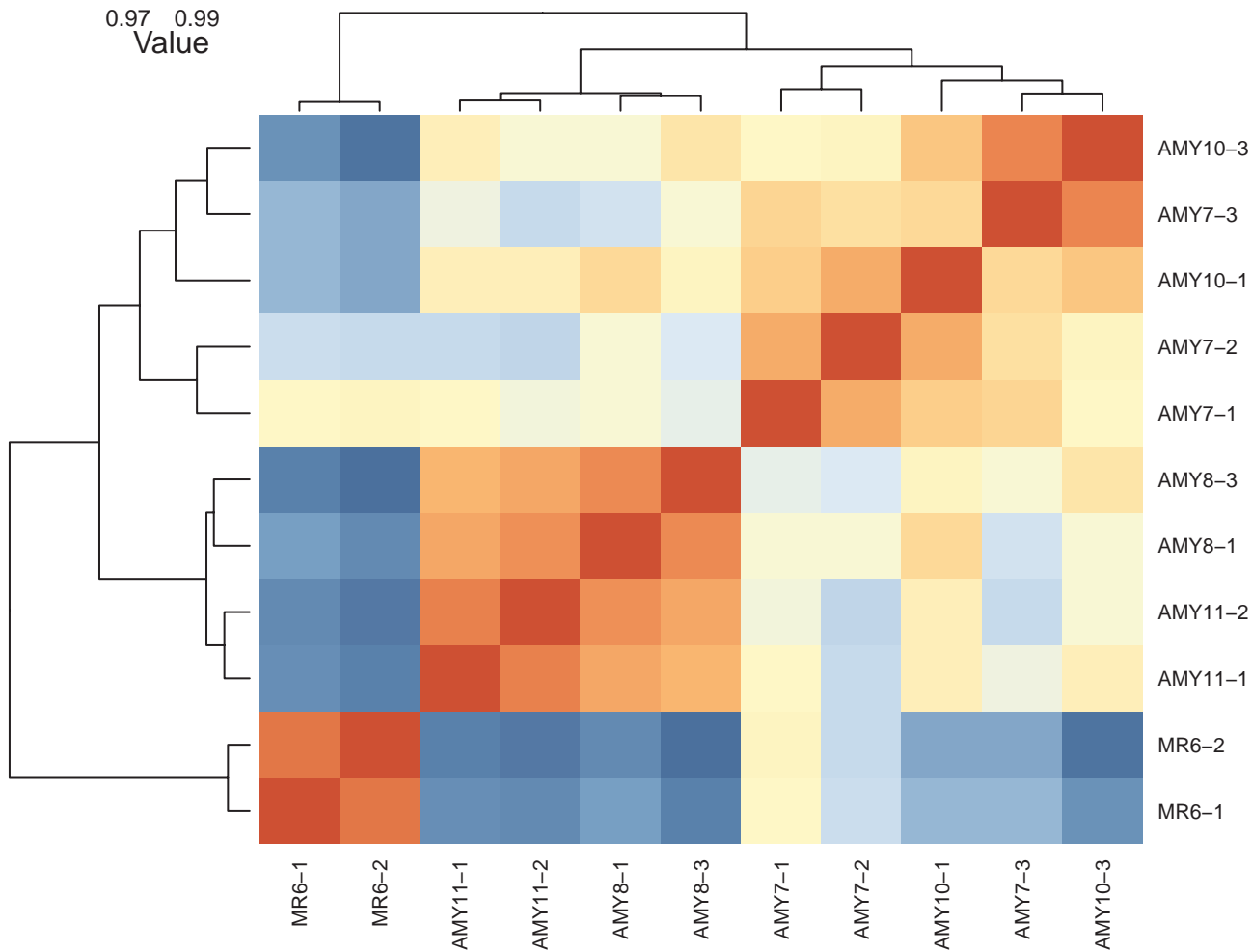

Supplement: Figure S4 — Correlation matrix demonstrating gene expression similarities among ATP9 relocation strains. Clustering reveals that greater gene expression differences in the relocation strains are induced by gene (PaAtp9-5 vs. PaAtp9-7) than by plasmid type (centromeric vs. multicopy), and that the biological replicates for each strain (indicated by numbers) display strong reproducibility. Samples were clustered hierarchically using the Euclidean distance between the pairwise Pearson correlation coefficients, which were computed between vectors of all per-gene normalized intensities per sample. Strains were grown in rich ethanol/glycerol media. (PDF) [file pgen.1002876.s005.pdf]
