# Supplementary material for: Experimental Relocation of the Mitochondrial ATP9 Gene to the Nucleus Reveals Forces Underlying Mitochondrial Genome Evolution
Source: PLoS Genet. 2012 Aug 16;8(8):e1002876. doi: 10.1371/journal.pgen.1002876 (PMC3420929; doi:10.1371/journal.pgen.1002876)
Supplement: Figure S3 — Construction of P. anserina ATP9 gene cassettes. A) Sequence of PaAtp9-7 cDNA cloned into pFL61 (pMB6C). When this plasmid was transformed into the Δatp9 strain RKY26, the resulting strain did not grow on glycerol media, but accumulated revertants following several rounds of selection. We isolated a revertant (strain MBE2) and sequenced the plasmid insert, revealing a precise deletion of the 5′ UTR (boxed sequence). We thus used synthetic genes optimized for yeast expression in subsequent experiments. B) Sequence of a synthetic version of PaAtp9-5 optimized for expression in yeast, cloned into pCM189 (pAM16) or pCM190 (pAM19). C) Sequence of a synthetic version of PaAtp9-7 optimized for expression in yeast, cloned into pCM189 (pAM17) or pCM190 (pAM20). D) Sequence of the hybrid/chimeric gene (Atp9-Hyb) encoding the MTS and first transmembrane segment of PaAtp9-7, followed by the connecting loop and second transmembrane segment of the protein encoded by yeast ATP9 (pAM12). Capitalized bases indicate coding sequences. Restriction sites used for cloning are underlined. (PDF) [file pgen.1002876.s004.pdf]

A

yAtp9-Nuc (plasmid pAM11)

*ggatcc***ATGAATGCCTCTTCTAAGCTGGCCGGTGCTGTTGCCAGAATGGGGGCGAAACCAGCTATGGCACAAACA**
AGTAGACTTCCATCATCTATCTTAAGGTCGACATCTATTGCGGGCCGTCATGGTTTGTTATTATCTCAGGGAAGG
AATGCTTTTGCGCCTGTTATGATGAGGTCGGCCACTCAATCAAGAGGTGTCGTAGCAGAAACTGCCATGCAATTA
GTCTTAGCCGCTAAGTACATAGGAGCAGGTATAAGCACTATTGGTTTATTGGGAGCGGGTATTGGGATTGCTATC
GTTTTTGCTGCCTTAATTAACGGTGTGTCTAGAAATCCCTCTATCAAGGACACTGTTTTTCCAATGGCAATCCTT
GGCTTTGCATTAAGTGAAGCAACAGGCCTTTTCTGTTTGATGGTTAGCTTCCTTCTGCTATTCGGAGTCTAA*ctg*
cag

B

PaAtp9-5 (plasmids pAM16 and PAM19)

*ggatcc***ATGGCATCTACCAGAGTATTAGCCTCACGTCTTGCATCTCAAATGGCAACTAAAGTAGCCAGACCTGCT**
GTCAGAGTTCCTGCAAGGGCCTTCACGGCAGGTACCAAGGCAACGCCACTTCAAGCTGTAAAGAGACAGCAAATG
TCATCCATTATCACGGCTACTAGACAGATTACGCAAAAGAGGGCCTACTCAAGTGAAATCGCACAAGCTATGGTT
GAAGTATCTAAGAACTTAGGGATGGGTACGGCGGCTATAGGTCTAACAGGTGCGGGTATAGGTATAGGGCTTGTA
TTTGCAGCTCTTCTAAATGGAGTTGCTAGAAATCCTGCCTTAAGAGGTCAACTGTTCTCCTACGCAATTTTAGGT
TTCGCTTTCGTAGAGGCTATCGGGTTATTCGATTTGATGGTAGCACTGATGGCGAAATTTACCTAG*ctgcag*

C

PaAtp9-7 (plasmids pAM17 and pAM20)

*ggatcc***ATGAACGCAAGTAGTAAATTAGCAGGCGCTGTCGCTAGAATGGGGGCCAAGCCAGCAATGGCTCAAACT**
TCTCGTCTGCCCAGTAGCATACTTCGTTCTACGTCGATTGCAGGAAGGCATGGATTATTGTTATCACAAGGCAGA
AATGCCTTCGCCCCTGTCATGATGAGAAGCGCAACACAGTCCAGAGGCGTCGTTGCCGAGACAGCCACTGCCGCT
ATTCTGGCGGCAGGAAAAATGCAAGGTGCTGGTTTGGCGACTATAGGTCTGTCTGGAGCTGGAGTAGGTATTGGT
ACTGTTTTTGCCGCCTTGATAAATGGAACGGCTAGAAACCCAGCGTTGAGGAGTCAGTTATTCAGTTATGCTATT
CTAGGTTTCGCTTTCGCCGAGGCAACAGGTCTGTTCGCTCTAATGGTCGCATTCCTTTTGCTATTTGCTTACTAG
ctgcag

D

Atp9-Hyb (plasmid pAM12)

*ggatcc***ATGAACGCTTCTTCAAAACTGGCTGGAGCTGTTGCTAGAATGGGTGCTAAACCTGCCATGGCACAGACT**
AGCAGATTGCCTTCCTCCATCCTGAGGTCTACTTCCATAGCAGGTAGACACGGATTGTTGCTATCCCAGGGCCGT
AATGCATTTGCACCCGTGATGATGCGTTCTGCCACACAAAGTAGGGGAGTAGTCGCTGAAACAGCTACAGCGGCA
ATTCTTGCCGCTGGCAAAATGCAAGGGGCAGGTCTTGCAACAATTGGATTGTCTGGCGCTGGTGTTGGGATTGGG
ACTGTGTTTGCAGCGCTTATAAATGGTGTATCAAGAAATCCATCCATAAAAGATACGGTCTTTCCAATGGCCATT
TTAGGATTCGCTTTATCAGAAGCTACTGGTTTATTTTGTTTGATGGTGAGCTTTCTATTGTTATTCGGCGTTTGA
ctgcag
