# Supplementary material for: Experimental Relocation of the Mitochondrial ATP9 Gene to the Nucleus Reveals Forces Underlying Mitochondrial Genome Evolution
Source: PLoS Genet. 2012 Aug 16;8(8):e1002876. doi: 10.1371/journal.pgen.1002876 (PMC3420929; doi:10.1371/journal.pgen.1002876)
Supplement: Table S1 — Plasmids used in this study. (DOCX) [file pgen.1002876.s006.docx]

**Table S1 – Plasmids used in this study**

| Name | Insert | Source |
| --- | --- | --- |
| pDS24 |  | [1] |
| Yep351 |  | ATCC |
| pJM2 |  | [2] |
| pMOS |  | GE Healthcare |
| pFL61 |  | [3] |
| pCM189 |  | ATCC |
| pCM190 |  | ATCC |
| pJ204 |  | DNA 2.0 |
| pRK1 | *atp9::ARG8^m^* cloned in pJM2 | This study |
| pJM2-ATP9 | *ATP9* cloned in pJM2 | This study |
| pMOS-ATP9 | *ATP9* cloned in pMOS | This study |
| pAM13 | *PaAtp9*-5 cloned in pJ204 | This study |
| pAM14 | *PaAtp9*-7 cloned in pJ204 | This study |
| pAM5 | y*Atp9*-Nuc cloned in pJ204 | This study |
| pAM6 | *Atp9*-Hyb cloned in pJ204 | This study |
| pAM16 | *PaAtp9*-5 cloned in pCM189 | This study |
| pAM17 | *PaAtp9*-7 cloned in pCM189 | This study |
| pAM19 | *PaAtp9*-5 cloned in pCM190 | This study |
| pAM20 | *PaAtp9*-7 cloned in pCM190 | This study |
| pAM11 | y*Atp9*-Nuc cloned in pCM190 | This study |
| pAM12 | *Atp9*-Hyb cloned in pCM190 | This study |

1. Steele DF, Butler CA, Fox TD (1996) Expression of a recoded nuclear gene inserted into yeast mitochondrial DNA is limited by mRNA-specific translational activation. Proc Natl Acad Sci U S A 93: 5253-5257.

2. Bonnefoy N, Fox TD (2001) Genetic transformation of Saccharomyces cerevisiae mitochondria. Methods Cell Biol 65: 381-396.

3. Minet M, Dufour ME, Lacroute F (1992) Complementation of Saccharomyces cerevisiae auxotrophic mutants by Arabidopsis thaliana cDNAs. Plant J 2: 417-422.
