# Supplementary material for: Experimental Relocation of the Mitochondrial ATP9 Gene to the Nucleus Reveals Forces Underlying Mitochondrial Genome Evolution
Source: PLoS Genet. 2012 Aug 16;8(8):e1002876. doi: 10.1371/journal.pgen.1002876 (PMC3420929; doi:10.1371/journal.pgen.1002876)
Supplement: Text S1 — Supplementary methods. (DOC) [file pgen.1002876.s007.doc]

**Supporting Information**

Experimental Relocation of the Mitochondrial *ATP9* Gene to the Nucleus Reveals Forces Underlying Mitochondrial Genome Evolution

Maïlis Bietenhader, Alexandre Martos, Emmanuel Tetaud, Raeka S. Aiyar, Carole H. Sellem, Roza Kucharczyk, Sandra Clauder-Münster, Marie-France Giraud, François Godard, Bénédicte Salin, Isabelle Sagot, Julien Gagneur, Michelle Déquard-Chablat, Véronique Contamine, Sylvie Hermann-Le Denmat, Annie Sainsard-Chanet, Lars M. Steinmetz and Jean-Paul di Rago

**Text S1. Supplementary Methods.**

**Yeast cell culture.** The following media were used for growing yeast: Rich media, YPGA: 1% (w/v) yeast extract, 1% (w/v) peptone, 2% (w/v) glucose, and 40 mg/L adenine); N3: 1% (w/v) yeast extract, 1% (w/v) peptone, 2% (w/v) glycerol, 50 mM potassium phosphate pH 6.2, and 40 mg/L adenine; YPGALA: 1% (w/v) yeast extract, 1% (w/v) peptone, 2% (w/v) galactose, and 40 mg/L adenine; N3: 2% glycerol, 1% yeast extract, 1% bactopeptone, 20 mg/ml adenine, 50 mM phosphate buffer, pH 6.2; YPEG: 2% (w/v) glycerol, 3% (w/v) ethanol, 1% (w/v) yeast extract, 1% (w/v) bactopeptone, 40 mg/L adenine. Minimal medium (WO): (0.17% (w/v) yeast nitrogen base without amino acids and without ammonium sulfate, 0.5% ammonium sulfate, 2% glucose or galactose and other supplements depending on the strain’s auxotrophic markers. Complete synthetic medium (CSM): 0.17% (w/v) yeast nitrogen base without amino acids and without ammonium sulfate, 0.5% (w/v) ammonium sulfate (w/v), 2% glucose or galactose, and 0.8% (w/v) of a CSM mixture of amino acids and bases (BIO-101). Solid media contained 2% (w/v) agar.

**Deletion of the yeast mitochondrial *ATP9* gene**. The plasmid pDS24 containing *ARG8^m^* was used for PCR amplification of an *atp9::ARG8^m^* DNA cassette with primers OligoproATP9 (5'-AAAAgaattcATGCAATTAGTATTAGCAGCTAAATATATTGGAGCAGGTATCTCAACA**ATGACACATTTAGAAAGAAGTAG**) and OligotermATP9 (5'- AAAAtctagaTTATACACCGAATAATAATAAGAATGAAACCATTAAACAGAATAAACCTGTA**TTAAGCATATACAGCTTCGATAGC**). Near the 5' ends of these primers are restriction sites (in lower-case letters) for *EcoRI* (in OligoproATP9) and *XbaI* (in OligotermATP9) that are followed by sequences homologous to *ATP9* (capital letters) and *ARG8^m^* (capital letters in bold). The resulting PCR product is designed to delete and replace a 131 bp region internal to the coding sequence of *ATP9* with *ARG8^m^* from nucleotides 49 to 179. After digestion with *XbaI* and *EcoRI*, the product was cloned into the plasmid pJM2 that contains *COX2* as a genetic marker for mitochondrial transformation . The resulting plasmid (pRK1) was introduced by co-transformation with the nuclear selectable *LEU2* plasmid (Yep351, from ATTC) into the ρ^0^ strain DFS160 by microprojectile bombardment using a biolistic PDS-1000/He particle delivery system (Bio-Rad) as previously described . Mitochondrial transformants (RKY4) were identified among the Leu^+^ nuclear transformants by their ability to produce respiring clones when mated to the non-respiring NB40-3C strain bearing a deletion in *COX2* . The transformants were purified by subcloning and crossed to MR6 , producing arginine prototrophs with the MR6 nucleus, of which some (RKY26) harbored the expected replacement of *ATP9* with *ARG8^m^*, as confirmed by DNA sequencing and the experiments shown in Figures 1 and S1.

**Construction of the ρ^-^ synthetic *ATP9* strains FG9 and MB2** –The wild-type *ATP9* locus, from position -228 upstream of the initiator codon to position +98 downstream of the stop codon, was PCR-amplified from strain SDC17-31b mtDNA using primers 5'-AATAAGATATATAAATAAGTCCC and 5'-GAATGTTATTAATTTAATCAAATGAG. The PCR product was blunt-end ligated into the *EcoRV* site of pMOS (pMOS Blue Blunt Ended Cloning Kit from GE Healthcare), yielding plasmid pMOS-ATP9. The *ATP9* insert was removed by digestion with *Bam* HI + *Xba* I and ligated to pJM2 cut with the same enzymes, creating plasmid pJM2-*ATP9*. The ρ^0^ strain DFS160-a was bombarded with this plasmid and plasmid Yep351. Mitochondrial transformants (FG9) were identified among Leu^+^ nuclear transformants by their ability to produce respiring clones when mated to YTMT2, a cytoductant of NB40-3C into DFS160. The mtDNA of FG9 was transferred via cytoduction into MR6-α (obtained from MR6 by HO-mediated mating type switching), to yield the strain MB2.

**Deletion of *YME1* in strain AMY5**. To delete *YME1* in the strain AMY5 (Δatp9 + y*Atp9*-Nuc), we PCR-amplified a null allele of the gene (yme1::KanMX4) from the Euroscarf strain Y17144 using the primers 5’- CCTTTTACGTTTCTGGTCGG and 5- GGGATGAAAGAAATTAATAACCC. In the resulting PCR product the KanMX4 gene is flanked by the 356 bp upstream and 291 bp downstream of the *YME1* coding sequence. Cells from AMY5 were transformed with 5 μg of the PCR product and plated on CSM medium lacking arginine and uracil supplemented with 200 mg per liter of G418. Clones resistant to the drug were tested for the *YME1* deletion by PCR and by measuring their sensitivity to ethidium bromide at 36°C (a typical phenotype conferred by the loss of *YME1* ).

**Construction of *P. anserina* strains for purification of ATP synthase**. Using *P. anserina* genomic DNA, three PCR products were generated: PCR1, with primers catcatcatcatcatCATTAAAGTAGACGACGCTGTT (His tag) + gggaagctTCTACTGATTCCCAACGTATC (*HindIII* site); PCR2, with primers ctttaatgatgatgatgatgATGGGAAGCCTTGGCGTTG (His tag) and gcaccaCCATGGCTCCCTTCTTCGC (*NcoI* site); PCR3, with primers agggagCCATGGTGGTGCTGCAAAAG (*NcoI* site) and gggagatctGGTTACATGTCTCTGTTGCTG (*Bgl* II site). The PCR1 and PCR2 products were fused by amplification with primers gcaccaCCATGGCTCCCTTCTTCGC and gggaagctTCTACTGATTCCCAACGTATC. The resulting fragment was fused to PCR3 with primers gggagatctGGTTACATGTCTCTGTTGCTG and gggaagctTCTACTGATTCCCAACGTATC, and the fusion product was cut with *BglII* and *HindIII* and cloned in the papi508 vector containing the *nat1* cassette. The resulting plasmid was linearized by *NcoI* and used for transforming protoplasts of a Δku70 strain (this strain is competent for *in vivo* homologous recombination, see . Nourseothricin-resistant clones were isolated and the correct integration of the modified subunit *j* gene was verified by PCR and sequencing for the correct integration of the modified *j* subunit gene. They were then crossed with either a Δ5 ^gpd^7^gpd^ strain lacking the native *PaAtp9-5* gene and carrying an ectopic *PaAtp9-7* gene under control of the 5’- and 3’-UTR sequences of *PaGpd* gene, or a Δ7 strain lacking the *PaAtp9-7* gene . The progeny of the crosses produced the expected strains with the His-tagged *j* subunit gene and where only *PaAtp9-5* or *PaAtp9-7* is expressed.

**Purification of ATP synthase from the *P. anserina* strains expressing either *PaAtp9-7* or *PaAtp9-5***. Mitochondria were prepared from strains of *P. anserina* expressing either *PaAtp9-5* or *PaAtp9-7* and where a (His)_6_-tag was added to the C-terminus of subunit *j* (the equivalent of the yeast subunit *i*), using a protocol adapted from . Two-day-old cultures on 30 Petri dishes were used to inoculate 60 flasks of liquid MTR rich medium . Mycellium (500 g) was collected after two days of incubation and treated for 3 hours with 30 g of glucanex in TPS1 buffer. Protoplasts were harvested by gaze filtration and washed successively with 0,66M saccharose (in 50mM Phosphate buffer), 0,6M sorbitol and 0,8M sorbitol (in 0,1M Tris-HCl pH 7,5). Mitochondria were released by osmotic shock in 0,33M saccharose in a potter followed by differential centrifugation, and were frozen as small beads in liquid nitrogen. A purification protocol, adapted from the one used to prepare yeast ATP synthase, was developed to isolate the two ATP-synthase isoforms of *P. anserina*. Submitochondrial particles (SMP) were prepared from the mitochondria according to (MacLennan et al. 1968) and centrifuged at 48000 *g* for 45 min at 4°C. Pellets were suspended at 10 mg of starting mitochondrial proteins per mL of buffer "A" (150 mM potassium acetate, 10% (V/V) glycerol, 2 mM PMSF, 2 mM ε-ACA, 30 mM HEPES pH 7.4 and EDTA-free protease inhibitor cocktail) containing 0.75% H_12_-TAC (w/v). After 30 min of incubation at 4°C, the extract was clarified by centrifugation (30 min, 4°C, 25000 *g*). The supernatant was diluted with two volumes of washing buffer "W" (50 mM NaCl, 10% (v/v) glycerol, 10 mM imidazole, 20 mM sodium phosphate pH 7.9) and mixed with Ni-NTA-agarose beads (Qiagen) (0.25 mL of slurry per 10 mg of starting mitochondrial proteins) pre-equilibrated in buffer "W". After an overnight incubation at 4°C, beads were washed with 25 volumes of buffer "W" containing 0.1% H_12_ –TAC (w/v). ATP synthase was eluted with buffer "E" (50 mM NaCl, 10% (v/v) glycerol, 250 mM imidazole, 0.1 % H_12_-TAC, 20 mM sodium phosphate pH 7.9). Enzyme purity was assessed by silver staining on a 12% Tricine SDS-PAGE.

**Respiratory and ATP synthesis/hydrolysis activities of isolated mitochondria**. ATPase activity was assayed by measuring the release of inorganic phosphate from ATP at 37°C in the presence or absence of oligomycin at pH 8.4 . Oxygen consumption rates were measured with a Clark electrode in respiration buffer (0.65 M mannitol, 0.36 mM EGTA, 5 mM Tris phosphate, 10 mM Tris maleate pH 6.8) as previously described . For ATP synthesis rate measurements, mitochondria (0.3 mg.ml^-1^) were placed in a 2-ml thermostatically controlled chamber at 28°C in respiration buffer. The reaction was started by the addition of 4 mM NADH, 1 mM ADP and stopped by adding 3.5% perchloric acid, 12.5 mM EDTA. Samples were then neutralized to pH 6.5 with KOH and 0.3 M MOPS. ATP was quantified by luciferin/luciferase assay (ThermoLabsystems) on an LKB bioluminometer.

**Northern and Southern blot analyses**. RNA was separated on a horizontal 1% (w/v) agarose, 6% (v/v) formaldehyde gel in MOPS buffer (20 mM MOPS; 5 mM sodium acetate; 1 mM EDTA), and transferred to a Nytran membrane (Schleicher & Schuell). The membrane was hybridized with specific radiolabelled DNA probes in 50% (v/v) formamide, 5x SSPE, 0.5% SDS, 7% PEG-5000, 5x Denhardt’s solution, 100 μg/ml carrier DNA at 42 °C. For the Southern blot analyses, mitochondrial DNA was digested with restriction enzymes, electrophoresed on a horizontal 0.8% agarose gel in 1X TAE buffer (Tris-acetate 20 mM, 50 mM EDTA pH 8), transferred to a Nytran membrane (Schleicher & Schuell), and hybridized with specific DNA probes in 6x SSC, 1x Denhardt’s solution, 0.1% SDS, 100 μg/ml carrier DNA at 60°C. The DNA probes were as follows: for *COX1*, plasmid pYGT21 ; for 15sRNA, plasmid pYJL23 (provided by J. Lazowska); for *ATP9*, a PCR product amplified from strain FY1679 mtDNA with primers 5'-tatgcaattagtattagcagc and 5'-GAATGTTATTAATTTAATCAAATGAG; for *ATP6*, a PCR product amplified from strain FY1679 mtDNA with primers 5'-GTATGATTCCATATTCATTTG and 5'-ATGTATCTTTTAAGTATGATGCTG; and for *ARG8^m^*, a PCR product amplified from plasmid pRK1 with primers 5'-ATGACACATTTAGAAAGAAGTAGA and 5'-TTAAGCATATACAGCTTCGATAGC . The DNA probes were labelled with [α^32^P]dCTP using the Rediprime II Kit and purified on MicroSpin^Tm^ G-25 columns from Amersham Biosciences.

**Transcription Profiling***.* The strains MR6, AMY7, AMY8, AMY10, and AMY11 were grown in rich ethanol-glycerol media (YPEG). Two biological replicates of each strain were cultured, harvested and hybridized to whole-genome tiling arrays as previously described, except that polyA RNA was isolated prior to cDNA synthesis (Couplan et al., PNAS 2011). Raw tiling array data was processed to provide normalized intensity values for each probe in each hybridization. The expression level of each transcript was estimated by the median value of the probe intensities of the transcript across both arrays per strain and condition. Transcripts were called expressed if their expression level was above background levels according to a previously described procedure . The distribution of background microarray signal intensities was estimated from the intensities of the probes outside transcript boundaries. The cut-off for an expression level to be significantly above background was then set at an estimated false discovery rate (FDR) of 0.1. Next, significance for differential expression between conditions of interest was determined using limma’s moderated t-test and corrected for multiple testing using the Benjamini-Hochberg method . Genes differentially expressed in glycerol were selected according to a FDR < 0.1 and a fold-change > 1.5 in either direction. Functional enrichments were calculated using the Ontologizer tool on Gene Ontology and transcription factor targets . Data can be browsed at <http://steinmetzlab.embl.de/atp9>, results of the analysis are in Dataset S1, and raw data was deposited in the ArrayExpress database (http:// www.ebi.ac.uk/arrayexpress/, accession number E-MTAB-1115).

**References**

Ashburner M, Ball CA, Blake JA, Botstein D, Butler H, Cherry JM, Davis AP, Dolinski K, Dwight SS, Eppig JT et al. 2000. Gene ontology: tool for the unification of biology. The Gene Ontology Consortium. *Nat Genet* **25**: 25-29.

Bauer S, Gagneur J, Robinson PN. 2010. GOing Bayesian: model-based gene set analysis of genome-scale data. *Nucleic Acids Res* **38**: 3523-3532.

Benjamini Y, Drai D, Elmer G, Kafkafi N, Golani I. 2001. Controlling the false discovery rate in behavior genetics research. *Behav Brain Res* **125**: 279-284.

Bonnefoy N, Fox TD. 2001. Genetic transformation of Saccharomyces cerevisiae mitochondria. *Methods Cell Biol* **65**: 381-396.

David L, Huber W, Granovskaia M, Toedling J, Palm CJ, Bofkin L, Jones T, Davis RW, Steinmetz LM. 2006. A high-resolution map of transcription in the yeast genome. *Proc Natl Acad Sci U S A* **103**: 5320-5325.

Déquard-Chablat M, Sellem CH, Golik P, Bidard F, Martos A, Bietenhader M, di Rago JP, Sainsard-Chanet A, Hermann-Le Denmat S, Contamine V. 2011. Two Nuclear Life Cycle-Regulated Genes Encode Interchangeable Subunits c of Mitochondrial ATP Synthase in Podospora anserina. *Mol Biol Evol* **28**: 2063-2075.

Duvezin-Caubet S, Caron M, Giraud MF, Velours J, di Rago JP. 2003. The two rotor components of yeast mitochondrial ATP synthase are mechanically coupled by subunit delta. *Proc Natl Acad Sci U S A* **100**: 13235-13240.

El-Khoury R, Sellem CH, Coppin E, Boivin A, Maas MF, Debuchy R, Sainsard-Chanet A. 2008. Gene deletion and allelic replacement in the filamentous fungus Podospora anserina. *Curr Genet* **53**: 249-258.

Foury F, Roganti T, Lecrenier N, Purnelle B. 1998. The complete sequence of the mitochondrial genome of Saccharomyces cerevisiae. *FEBS Lett* **440**: 325-331.

Godard F, Tetaud E, Duvezin-Caubet S, di Rago JP. 2011. A genetic screen targeted on the FO component of mitochondrial ATP synthase in Saccharomyces cerevisiae. *J Biol Chem* **286**: 18181-18189.

Guerin B, Labbe P, Somlo M. 1979. Preparation of yeast mitochondria (Saccharomyces cerevisiae) with good P/O and respiratory control ratios. *Methods Enzymol* **55**: 149-159.

MacIsaac KD, Wang T, Gordon DB, Gifford DK, Stormo GD, Fraenkel E. 2006. An improved map of conserved regulatory sites for Saccharomyces cerevisiae. *BMC Bioinformatics* **7**: 113.

Rak M, Tetaud E, Godard F, Sagot I, Salin B, Duvezin-Caubet S, Slonimski PP, Rytka J, di Rago JP. 2007. Yeast cells lacking the mitochondrial gene encoding the ATP synthase subunit 6 exhibit a selective loss of complex IV and unusual mitochondrial morphology. *J Biol Chem* **282**: 10853-10864.

Rigoulet M, Guerin B. 1979. Phosphate transport and ATP synthesis in yeast mitochondria: effect of a new inhibitor: the tribenzylphosphate. *FEBS Lett* **102**: 18-22.

Smyth GK, Yang YH, Speed T. 2003. Statistical issues in cDNA microarray data analysis. *Methods Mol Biol* **224**: 111-136.

Somlo M. 1968. Induction and repression of mitochondrial ATPase in yeast. *Eur J Biochem* **5**: 276-284.

Steele DF, Butler CA, Fox TD. 1996. Expression of a recoded nuclear gene inserted into yeast mitochondrial DNA is limited by mRNA-specific translational activation. *Proc Natl Acad Sci U S A* **93**: 5253-5257.

Szczepanek T, Lazowska J. 1996. Replacement of two non-adjacent amino acids in the S.cerevisiae bi2 intron-encoded RNA maturase is sufficient to gain a homing-endonuclease activity. *EMBO J* **15**: 3758-3767.

Weber ER, Rooks RS, Shafer KS, Chase JW, Thorsness PE. 1995. Mutations in the mitochondrial ATP synthase gamma subunit suppress a slow-growth phenotype of yme1 yeast lacking mitochondrial DNA. *Genetics* **140**: 435-442.
